# The effects of lingual training: a systematic review with meta-analysis

**DOI:** 10.1590/2317-1782/20232021324en

**Published:** 2023-08-21

**Authors:** Nathalya de Faria Fonseca, Andréa Rodrigues Motta, Fernanda Campos de Freitas, Mariana Rezende Nonato, Elton Mendes Francelino, Renata Maria Moreira Moraes Furlan

**Affiliations:** 1 Universidade Federal de Minas Gerais - UFMG - Belo Horizonte (MG), Brasil.; 2 Universidade Federal da Bahia - UFBA - Salvador (BA), Brasil.

**Keywords:** Tongue, Exercise Therapy, Myofunctional Therapy, Rehabilitation, Speech, Language and Hearing Sciences

## Abstract

**Purpose:**

To assess the effectiveness of myotherapy exercises in increasing tongue pressure and strength. A secondary aim was to analyze the exercise types, training parameters, and functional results.

**Research strategies:**

This systematic literature review was based on the Prisma protocol guidelines.

**Selection criteria:**

The review included clinical trials that assessed the effects of tongue muscle training, with no restriction on the language or year of publication.

**Data analysis:**

The steps included eliminating duplicates; reading abstracts and excluding studies that did not meet the inclusion criteria; reading selected articles in full text, extracting important data, and gathering them in a table; and meta-analysis, using the inverse variance method. The methodological quality of the studies was assessed with the Joanna Briggs Institute’s tool. The quality of evidence was assessed with the Grading System of Recommendations Assessment, Development and Evaluation.

**Results:**

The meta-analysis indicated a significant increase in maximum anterior and posterior pressure as an effect of training. The most performed exercise was tongue pressure against the palate. However, training parameters varied between studies, and whether exercises alone led to functional improvement cannot be stated. The quality of the evidence was considered low.

**Conclusion:**

Myotherapy exercises increased anterior and posterior tongue pressure in adults, but the quality of this evidence is low. The studies used various exercise types and training parameters. It cannot be stated whether exercises alone led to functional improvement.

## INTRODUCTION

The tongue, which is involved in all functions of the stomatognathic system, is essential to the nutrition and communication process and occlusion stability^(1)^. Its structure is characteristic of unique organisms called muscular hydrostats, which also include the trunks of elephants and tentacles of octopuses^(2,3)^. These organs are made exclusively of muscles that can make and sustain various movements thanks to their fibers, which are oriented in various directions: longitudinal, vertical, transversal, and, in some cases, circular^(2,3)^.

The tongue has intrinsic and extrinsic muscles, whose different groups interact to carry out its functions - most movements require intense and simultaneous contraction of various groups^(4)^. Types I and IIa muscle fibers predominate in the anterior portion of tongue morphology. Type I fibers are resistant to fatigue, while type IIa fibers contract quickly. This combination favors speech movements, which are quick and repetitive and do not need much strength. The base of the tongue predominantly has type IIb fibers, which can generate greater strength, important for swallowing^(5)^.

Given all these specificities, it may not be a good option to treat changes in tongue strength and/or resistance by applying exercise physiology based on the same principles used for the other body muscles. Exercise-based therapy, called myotherapy, is used to rehabilitate and/or prevent orofacial muscle changes. It belongs to the area of oral-motor control as part of speech-language-hearing practices, aiming to improve strength, resistance, mobility, and coordination^(6)^. Moreover, besides myotherapy and preferably associated with it, speech-language-hearing therapists can use orofacial myofunctional therapy to improve stomatognathic system structures and functions with assisted functional training^(7)^.

Researchers have been trying for some years to find methods to improve tongue muscle training; hence, many instruments and exercises have been developed to this end^(8)^. However, while the diversity of exercises broadens individualized treatment possibilities and positively impacts their effectiveness, it can also hinder the knowledge and development of such techniques if their effects are not addressed in studies. This article presents the results of an investigation on the effects of tongue training to reflect on its effectiveness in the perspective of speech-language-hearing care.

## PURPOSE

This research aimed to assess the effectiveness of myotherapy exercises to increase tongue pressure and strength. Secondarily, it aimed to analyze which exercise types and training parameters (contraction time, number of repetitions, amount of training per week, training duration) are used and their functional results.

## SEARCH STRATEGY

This systematic review of the literature was registered in the International Prospective Register of Systematic Reviews (PROSPERO) (CRD42021224324) and developed according to the Preferred Reporting Items for Systematic Reviews and Meta-Analyses (PRISMA)^(9)^. The review had the following stages: developing the research question, defining keywords and article eligibility criteria, selecting articles, and critically assessing them.

The research question for this study was as follows: “Do tongue myotherapy exercises increase its strength/pressure?”. Secondarily, the study sought to find the types, parameters, and functional effects of the exercises that are used.

Articles were selected by surveying the national and international literature, with no restriction on the language or year of publication, in the following databases: *Biblioteca Brasileira de Odontologia* (BBO - Brazilian Dental Library) via Virtual Health Library (VHL), CINAHL, Cochrane, EMBASE, LILACS (via VHL), MEDLINE (via PubMed), Scopus, and Web of Science. The descriptors were obtained from the Medical Subject Headings (MeSH), Health Sciences Descriptors (DeCS), and EMBASE Subject Headings (Emtree), as follows: tongue, muscle strength, physical endurance, resistance training, exercise therapy, rehabilitation, exercise, as well as the free terms: lingual and tongue strength, and their equivalents in Portuguese and Spanish. The search strategies are shown in Chart 1. All databases were searched in September 2020.

**Chart 1:** Search strategies per database

**Source**	**Search strategy**
VHL (BBO and LILACS)	(tongue OR lengua OR língua OR lingual) AND (“Muscle Strength” OR “Fuerza Muscular” OR “Força Muscular” OR “Physical Endurance” OR “Resistencia Física” OR “Resistência Física” OR “Força da Língua” OR “Tongue Strength” AND (“Resistance Training” OR “Entrenamiento de Resistencia” OR “Treinamento de Resistência” OR “Exercise Therapy” OR “Terapia por Ejercicio” OR “Terapia por Exercício” OR “Exercício Terapêutico” OR “Exercício de Reabilitação” OR rehabilitation OR rehabilitación OR reabilitação OR habilitação OR exercise OR “Ejercicio Físico” OR “Exercício Físico”) AND (db:(“LILACS” OR “BBO”))
CINAHL	(tongue OR lingual) AND (“muscle strength” OR “physical endurance” OR “tongue strength”) AND (“resistance training” OR “exercise therapy” OR rehabilitation OR exercise)
Cochrane	(tongue OR lingual) AND (“muscle strength” OR “physical endurance” OR “tongue strength”) AND (“resistance training” OR “exercise therapy” OR rehabilitation OR exercise)
EMBASE	tongue AND “muscle strength” OR “endurance” OR “tongue strength” AND “resistance training” OR rehabilitation OR exercise
PubMed	(tongue [MeSH Terms] OR lingual) AND (“muscle strength” [MeSH Terms] OR “physical endurance” [MeSH Terms] OR “tongue strength”) AND (“resistance training” [MeSH Terms] OR “exercise therapy” [MeSH Terms] OR rehabilitation [MeSH Terms] OR exercise [MeSH Terms])
Scopus	(ALL(tongue) OR ALL(lingual)) AND (ALL(“muscle strength”) OR ALL(“physical endurance”) OR ALL (“tongue strength”)) AND (ALL(“resistance training”) OR ALL (“exercise therapy”) OR ALL(rehabilitation) OR ALL(exercise)
Web of Science	ALL=((tongue OR lingual) AND (“muscle strength” OR “physical endurance” OR “tongue strength”) AND (“resistance training” OR “exercise therapy” OR rehabilitation OR exercise))

## SELECTION CRITERIA

Eligibility criteria were defined based on the PICOT elements: participants (individuals older than 18 years, with no restriction on sex or clinical condition); intervention (tongue strength/pressure or resistance training exercises); comparator (individuals who did not do the proposed exercises or underwent other therapeutic strategies); outcomes (strength/pressure and performance values in orofacial functions); type of study (randomized or nonrandomized clinical trials). After analyzing the titles and abstracts, the texts that were or could be compatible with the eligibility criteria were read in full text.

The inclusion criteria for article eligibility were as follows: original research articles designed as clinical trials; whose sample comprised individuals above 18 years old that were submitted to tongue muscle exercises; that had a comparator group comprising individuals who were not submitted to the approached exercises or underwent other therapeutic strategies; and that assessed as outcomes the strength or pressure values and/or orofacial function performance. The exclusion criteria were studies that did not address at least one of the following data: exercise type, training parameters, and results regarding at least one of the outcomes.

## DATA ANALYSIS

After reading the full text of the articles that met the eligibility criteria, their data were collected in a table developed to contain the following information: author, year of publication, the country where the study was conducted, characteristics of the sample, exercise type, training parameters, instruments used in data collection, and study results, emphasizing tongue pressure or strength values.

The methodological quality of these studies was assessed with JBI’s Critical Appraisal Checklist for Randomized Controlled Trial Studies^(10)^. This instrument presents criteria to assess the methodological quality of studies, with three possible answers: yes, this criterion is verified; no, this criterion is not verified; and it is unclear. Each positive answer scores 1 point and, the other ones score 0 points. The higher the score, the greater the internal quality and the smaller the risk of bias regarding the study’s methodological quality. It was determined that studies with less than 50% of positive answers would be considered as having low methodological quality; between 50 and 75% of positive answers, intermediate methodological quality; and with 75% or more positive answers, high methodological quality.

Publication bias was analyzed with funnel plots and the Egger test, using the STATA statistical program, version 13.0. The quality of evidence was assessed with the Grading of Recommendations Assessment, Development and Evaluation (GRADE)^(11)^.

All research stages were carried out by three researchers, who also conducted manually and independently the data analysis that determined whether studies met the eligibility criteria, using a binary scale (yes/no) and a Microsoft Excel^®^ spreadsheet. Articles assessed positively by two researchers were included in the study. Data were likewise extracted into a Microsoft Excel^®^ spreadsheet by at least one of the researchers and verified by at least one of the other ones. The quality of the studies was analyzed by one researcher and verified by another one.

The intervention effect measure considered for meta-analysis was the difference in anterior and posterior tongue pressure before and after the intervention, using the inverse variance method in STATA, version 13.0. The studies were analyzed both together and subdivided into clinical conditions and age.

## RESULTS AND DISCUSSION

**Figure 1:**
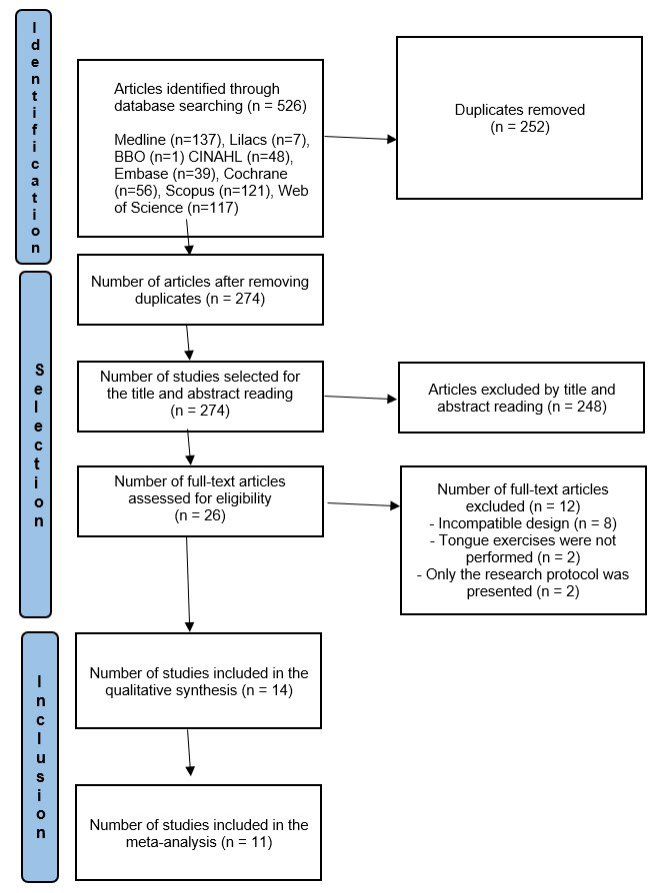
Flowchart with the various phases of the review based on the PRISMA protocol guidelines. Source: Flow Diagram^(9)^

The search in the databases initially found 526 references on tongue muscle training exercises (137 in MEDLINE, seven in LILACS, one in BBO, 48 in CINAHL, 39 in EMBASE, 56 in Cochrane, 121 in Scopus, and 117 in Web of Science). After removing the duplicates, 274 articles remained, and after excluding articles by abstract reading, 26 remained, which were read in full text. After excluding another 12 articles for not meeting the eligibility criteria, 14 articles reached the final inclusion phase for analysis, as shown in Figure 1.

The analysis of the studies included in this research readily showed that the interest in the topic is fairly recent, as they were published between 2003 and 2020. This may be explained by the also recent appearance of oral-motor control as a regulated speech-language-hearing practice. In Brazil, for example, speech-language-hearing therapy was regulated as a profession only in 1981, and titles of specialists, including oral-motor control, were regulated as late as 1996^(12)^. The very instruments used to measure tongue strength/pressure, despite their considerable number, are likewise recent, and some are still being improved^(8)^. Nevertheless, many countries are concerned with stomatognathic function rehabilitation. Two out of the 14 articles (all of them published in English) are Brazilian^(13,14)^, whereas South Korea published the most, with eight articles^(15-22)^; as for the other ones, two are from the United States^(23,24)^, one from China^(25)^, and one from Belgium^(26)^. The predominating age range in the samples comprised young adults and older adults, ranging from 24 to 85 years old, in an approximately even number of men and women.

Most studies approached people with dysphagia, four of them due to stroke^(15-17,19)^ and one due to oral cavity and/or oropharyngeal cancer, in a recent postoperative period from tumor resection surgery^(25)^. Changes in tongue strength/pressure can affect both the oral and pharyngeal phases of swallowing, and adequate strength must be used to ensure effective and safe swallowing^(17)^ - which explains the significant number of studies in this population included in this research. The review also included one study in people with post-stroke dysarthria^(18)^. The individuals in these studies had similar mean ages, ranging from 56.2 to 67.3 years. The main findings of the studies in individuals with dysphagia or dysarthria after stroke or mouth and/or oropharyngeal cancer are shown in Chart 2.

**Chart 2:** Summary of the main findings of the articles in individuals with orofacial changes (dysphagia or dysarthria after stroke and mouth cancer)

**Author (year of publication), country**	**Sample**	**Description of exercises**	**Treatment frequency and duration**	**Measuring method and outcomes assessed**	**Tongue pressure**	**Results**
Park et al. (2015)^(15)^	27 adults with post-stroke dysphagia.	-Tongue pressure against the palate using IOPI (tip and dorsum), maintaining 80% of 1 MR for 2 s.	10 series with 5 repetitions (anterior + posterior)/day, 5x/week, for 4 weeks,	-IOPI (maximum anterior and posterior pressure)	-Anterior pressure:	-Increase in the anterior portion in EG and CG.
South Korea	EG = 15 individuals, m = 67.3±10.6 years, 6 men (exercises + dysphagia therapy).		minimum 30-s intervals between repetitions.	-Videofluoroscopy	EG: 18.9±6.7 kPa (before) and 20.7±6.6 kPa (after);	-Increase in the posterior portion in EG.
	CG = 14 individuals, m = 65.8±11.5 years, 7 men (dysphagia therapy)				CG: 22±5.7 kPa (before) and 22.9±5.4 kPa (after).	-Improved oral phase of swallowing in EG and CG and pharyngeal phase of swallowing in EG.
					-Posterior pressure:	
					EG: 16.2±4.7 kPa (before) and 18.5±4.1 kPa: (after);	
					CG: 17.3±4.3 kPa (before) and 17.7±4.36 kPa (after).	
Byeon	48 adults with post-stroke dysphagia.	-Tongue protrusion, tongue lifting, and tongue lowering.	Exercises performed for 30 minutes a day, 5x/week, for 3 weeks.	-IOPI (lifting pressure and protrusion pressure, lip pressure and cheek pressure)	-Anterior tongue pressure	-Increased anterior tongue lifting pressure in EG and CG.
(2016)^(16)^	EG = 23 individuals, m = 62.5±6.5 years, 8 men (exercises + tactile thermal stimulation).	-Massage on the cheeks and neck, lip closure and protrusion, cheek inflation and sucking, and tongue protrusion, lifting, and lowering.		-Praat (diadochokinesia)	EG: 20.8±13.2 kPa (before) and 26.8±14.3 kPa (after)	-Improved diadochokinesia in EG.
South Korea	CG = 25 individuals, m = 64.1±7.1 years, 6 men (tactile thermal stimulation).				CG: 18.5±11.5 kPa (before) and 21.5±10.8 kPa (after).	
Kim et al. (2017)^(17)^	35 individuals with post-stroke dysphagia	-Tongue counter-resistance exercise against the palate (anterior and posterior region).	30x/day, 5x/week, for 4 weeks.	-IOPI (maximum anterior and posterior tongue pressure)	-Anterior pressure	-Increased anterior and posterior tongue pressure in EG in 4 weeks.
South Korea	EG1 = 18 individuals, m = 62.2±11 years, 11 men (tongue force exercises + traditional dysphagia therapy).			-Videofluoroscopy (functional swallowing assessment - Videofluoroscopic Dysphagia Scale and Rosenbek’s penetration-aspiration scale)	EG: 32.7±10.8 kPa (before) and 41.9±9.5 kPa (after);	-Increased anterior and posterior tongue pressure in relation to CG.
	CG = 17 individuals, m = 59.3±10.2, 8 men (traditional dysphagia therapy).				GC: 29.6±10.4 kPa (before) and 32.5±10.2 kPa (after).	-Improved oral and pharyngeal phases of swallowing in EG and CG.
					-Posterior pressure	-Improved oral and pharyngeal phases of swallowing EG in relation to CG.
					EG: 28.1±7.6 kPa (before) and 39.1±7.8 kPa (after)	-Improvement on the penetration-aspiration scale in EG and CG.
					CG: 26.6±9.1 kPa (before) and 31.4±9.7 kPa (after).	
Byeon	21 individuals with post-stroke dysarthria	EG and CG: pressing the IOPI bulb against the palate, exerting 50%, 75%, and 100% of the maximum tongue force; 4 series with 5 repetitions.	30min/day, 5x/week, for 4 weeks.	-IOPI (maximum tongue pressure);	-Anterior pressure	-EG had greater maximum tongue force than CG.
(2018)^(18)^	EG = 10 individuals, m = 65.85±9.23 years (tongue pressure exercises + tongue exercises).	EG: (i) Lifting the tip of the tongue and sustaining it for 5 s; (ii) lifting the tip of the tongue against a spatula and sustaining it for 5 s; (iii) lateralizing the tongue and sustaining it for 5 s to the right and 5 s to the left.		-Percentage of correctly articulated consonants.	EG: 10.7±9.8 kPa (before) and 20.8±16.9 kPa (after);	-No differences were found in the percentage of correctly articulated consonants between EG and CG.
South Korea	CG = 11 individuals, m = 67.03±7.60 (tongue pressure exercises)	(iv) lateralizing the tongue against a spatula and sustaining it for 5 s to the right and 5 s to the left; (v) protruding the tongue for 5 s and protruding the tongue against a spatula for 5 s.			CG: 11.4±8.1 kPa (before) and 17.9±15.1 (after).	
Hsiang et al. (2019)^(25)^	50 adults with oral cavity and/or oropharyngeal cancer submitted to tumor resection surgery during a recent post-operational period (48 men, m = 56.2±8.8 years).	-Tongue, lip, and mandible mobility exercises: Sustaining maximum structure extension for 1-2 s, then relaxing.	Exercises performed 10x/session,	-Videofluoroscopy (Rosenbek’s penetration-aspiration scale and oral cavity and pharyngeal residues)	Not assessed	-Improvement on the penetration-aspiration scale in EG.
China	EG = 25 (exercises)	-Tongue resistance exercises: counter-resistance against a spatula for 5 s.	3 sessions/day, for 12 weeks.			-Decreased amounts of nectar, honey, and pudding consistency residues in EG.
	CG = 25 (changes in body posture and food consistency)					
Park et al. (2019)^(19)^	24 adults with post-stroke dysphagia.	-Effortful swallowing training: pressing the tongue firmly against the palate while contracting the neck muscle and swallowing as strongly as possible.	Exercises performed 10x/session,	-IOPI (maximum anterior and posterior pressure)	-Anterior pressure:	-Increased anterior pressure in EG and CG.
South Korea	EG = 12 individuals, m = 66.5±9.5 years, 6 men (effortful swallowing + dysphagia therapy).		3 sessions/day, for 4 weeks.	-Videofluoroscopy (functional swallowing assessment - (Videofluoroscopic Dysphagia Scale)	EG: 20.8±4.3 kPa (before) and 27.6±4.3 kPa (after);	-Increased posterior pressure in EG and CG.
	CG = 12 individuals, m = 64.8±11.2 years, 5 men (dysphagia therapy)				CG: 21.2±5.8 kPa (before) and 23.1±5.4 kPa (after). -Posterior pressure:	-Improved oral and pharyngeal phases of swallowing in EG and CG.
					EG: 16.6±5.0 kPa (before) and 23.2±5.4 kPa (after);	
					CG: 16.7±4.4 kPa (before) and 18.2±4.5 kPa (after).	

**Caption:** CG = Control Group; EG = Experimental Group; IOPI = Iowa Oral Performance Instrument; m = Mean Age; x = Times; MR = Maximum Repetition; s = Seconds

Paying attention to tongue strength and its relationship with swallowing is more relevant among older adults, whose tone decreases due to the loss of muscle mass (which is inherent to aging) and reserve strength^(27)^, which makes them more vulnerable to dysphagia. This justifies that half of the studies in individuals without a history of orofacial changes addressed older adults^(21,22,26)^, while the other half comprised adults^(20,23,24)^. Charts 3 and 4 present the main findings of the studies in individuals without orofacial changes, respectively comprising adults and older adults.

**Chart 3:** Summary of the main findings of the articles in adults without orofacial changes

**Author (year of publication), country**	**Sample**	**Description of exercises**	**Treatment frequency and duration**	**Measuring method and outcomes assessed**	**Tongue pressure**	**Results**
Lazarus et al. (2003)^(23)^ United States	31 healthy individuals, m = 26 years.	-Pressing the tongue against a spatula or the IOPI bulb in the left and right directions, protrusion, and lifting, as strong as possible for 2 s.	10 repetitions, 5x/day, 5x/week, for 4 weeks.	-IOPI (maximum tongue pressure and resistance).	-Maximum anterior pressure:	-Increased maximum tongue pressure in EG1 and EG2.
	EG1 **=** 10 individuals, 2 men (strength exercise using a spatula).				EG1: 63.9±2.2 kPa (before) and 72.1±2.1 kPa (after);	-Increased tongue resistance in EG1 and EG2.
	EG2 **=** 10 individuals, 1 man (strength exercise using IOPI).				EG2: 64.8±3.0 kPa (before) and 74.0±2.4 kPa (after);	-Increased maximum tongue pressure when comparing the combined EG1 and EG2 results with CG.
	CG **=** 10 individuals, 5 men (no intervention)				CG: 69.8±5.6 kPa (before) and 71.2±5.4 (after).	
Clark (2012)^(24)^	25 individuals, m = 29.8 years; 3 men	EG1: pressing the tongue against the IOPI bulb on the palate with maximum strength.	1x/day, 3x/week, for 4 weeks	-IOPI (anterior tongue lifting pressure, resistance, power, and speed).	EG1: 65.8 kPa (before) and 82.6 kPa (after);	-Increased pressure in EG1, isotonic resistance in EG2, and power in EG3.
United States	EG1 = 5 individuals (strength training).	EG2: pressing against the IOPI bulb at 50% of the maximum strength as many times as possible (isotonic) and pressing the bulb at 50% of the maximum strength for as long as possible (isometric).			EG2: 65.6±15.2 kPa (before) and 73.0±18.4 kPa (after);	-Neither isometric resistance in EG2 nor speed in EG4 increased.
	EG2 = 5 individuals (resistance training).	EG3: repeating the phoneme /t/ as fast as possible, pushing the bulb at 5% of the maximum strength for 10 s.	EG1: 5 series of 5 repetitions.		EG3: 60.2±18.0 kPa (before) and 66.6±17.0 kPa (after);	
	EG3 = 5 individuals (power training).	EG4: repeating the phoneme /t/ as fast as possible for 10 s.	EG2: 5 series with 5% of the maximum number of repetitions.		EG4: 72.8±14.7 kPa (before) and 80.4±20.1 kPa (after);	
	EG4 = 5 individuals (speed training).		EG3: 5 series of 10 repetitions.		CG: 66.8±13.2 kPa (before) and 73.6±10.1 kPa (after).	
	CG = 5 individuals (no intervention).		EG4: five 10-s series.			
Park et al. (2019)^(20)^	30 healthy adults	-Tongue pressure against the palate with maximum strength (isotonic and isometric).	The isotonic exercise was performed 30x (2 s) and the isometric one 3x (10 s), 1x/day, 5x/week, for 6 weeks.	-IOPI (tongue pressure).	-Anterior pressure	-Increased tongue pressure in GE. -Increased tongue, mylohyoid, and digastric thickness in EG.
South Korea	EG = 15 individuals, m = 24.5±5.3 years, 8 men (exercise).			-Ultrasound (tongue, mylohyoid, and digastric thickness).	EG: 52.5±4.4 kPa (before) and 57.7±5.2 kPa (after);	-Increased tongue thickness in CG.
	CG = 15 individuals, m = 25.1±4.2 years, 7 men (no intervention).				CG: 53.8±3.0 kPa (before) and 54.7±1.95 kPa (after).	

**Caption:** CG = Control Group; EG = Experimental Group; IOPI = Iowa Oral Performance Instrument; m = Mean Age; x = Times; s = Seconds

**Chart 4:** Summary of the main findings of the articles in older adults without orofacial changes

**Author (year of publication), country**	**Sample**	**Description of the exercises**	**Treatment frequency and duration**	**Measuring method and outcomes assessed**	**Tongue pressure**	**Results**
Van den Steen et al. (2019)^(26)^	60 Older adults	-Pressing the IOPI bulb against the palate in the anterior and posterior positions:	60x anterior and 60x posterior, divided into 24 series of 5 repetitions with 30-s rest between them, for 3 non-consecutive days a week, for 8 weeks.	-IOPI (maximum anterior and posterior pressure) at the beginning of the study, after 4 and 8 weeks of training.	-Maximum anterior pressure:	-All three experimental groups had increased pressure in 4 weeks and 8 weeks.
Belgium	EG1 = 15 individuals, m = 79 years, 7 men	EG1: at 100% of 1 MR.			EG1: 36.9 ± 9.1 kPa (before) and 59.4±12.6 kPa (8 weeks)	-Greater maximum pressure in all EG than in CG.
	EG2 = 16 individuals, m = 81 years, 7 men	EG2: at 80% of 1 MR.			EG2: 34.1 ± 8.0 kPa (before) and 54.7±7.7 kPa (8 weeks)	
	EG3 = 16 individuals, m = 77 years, 3 men	EG3: at 60% of 1 MR.			EG3: 35.3 ± 6.8 kPa (before) and 53.6±7.31 kPa (8 weeks)	
	CG = 13 individuals	CG: pressing the IOPI bulb between the lips.			CG: 39.2 ± 9.9 kPa (before) and 44.5kPa± 11.7 (8 weeks)	
					-Maximum posterior pressure:	
					EG1: 30.2±8.3 kPa (before) and 52.7±12.3 kPa (8 weeks)	
					EG2: 34.0±7.6 kPa (before) and 51.1±9.9 kPa (8 weeks)	
					EG3: 32.8±4.4 kPa (before) and 50.3±8.1 kPa (8 weeks)	
					CG: 34.6±8.7 kPa (before) and 38.9±12.3 kPa (8 weeks)	
Park et al. (2019)^(21)^	40 older adults.	-Pressing the tongue against the palate at 70% of 1 MR (isotonic and isometric).	The isotonic exercise was performed 30x and the isometric one 3x (30 s), 3x/day. The training duration was not specified.	-IOPI (tongue pressure)	-Anterior pressure:	-Increased tongue pressure in EG and tongue, mylohyoid, and digastric thickness.
South Korea	EG = 20 individuals, m = 69.5±4.3; 10 men (exercise);			-Ultrasound (tongue, mylohyoid, and digastric thickness).	EG: 37.1±3.5 kPa (before) and 43.9±4.9 kPa (after);	-Unchanged tongue pressure and thickness in CG.
	CG = 20 individuals, m = 68.4±3.9 years; 11 men (no intervention).				CG: 36.6±3.3 kPa (before) and 37.1±3.4 kPa (after).	
Lee et al. (2020)^(22)^	74 older adults (m = 75 years)	EG1: (i) Swallowing saliva without sticking the tongue out of the mouth; (ii) swallowing saliva strongly without sticking the tongue out of the mouth; (iii) swallowing saliva with about one third of the tongue out of the mouth; and (iv) swallowing saliva with about two thirds of the tongue out of the mouth. Three repetitions each.	3x/day, 3x/week for 8 weeks.	-IOPI (anterior and posterior tongue pressure and lip pressure)	-Anterior pressure:	-Increased anterior and posterior tongue pressure in EG1.
South Korea	EG1 = 30 individuals, 3 men (swallowing with tongue control).	EG2: pressing the IOPI bulb between the tongue and the hard palate. 30 repetitions.		-Salivary flow rate	EG1: 34.3±10.1 kPa (before) and 38.5±13.4 kPa (after):	-Increased anterior tongue pressure in EG2.
	EG2 = 22 individuals, 3 men (resistance training with tongue pressure).			-Score of the Oral Health Impact Profile-14.	EG2: 40.6±11.5 kPa (before) and 45.5±11.0 (after);	-Increased salivary flow rate in EG1 and EG2.
	CG = 22 individuals, 6 men (no intervention).				CG: 39.1±12.9 kPa (before) and 38.4±11.1 kPa (after).	-Absence of impact in Oral Health Impact Profile-14 in the groups.
					-Posterior pressure:	
					EG1: 33.8±13.8 kPa (before) and 38.1±15.0 kPa (after);	
					EG2: 41.4±11.2 kPa (before) and 45.1±9.7 (after);	
					CG: 35.6±15.3 kPa (before) and 38.8±12.8 kPa (after).	

**Caption:** CG = Control Group; EG = Experimental Group; IOPI = Iowa Oral Performance Instrument; m = Mean Age; x = Times; MR = Maximum Repetition; s = Seconds

This research included two studies on primary snoring and/or obstructive sleep apnea (OSA)^(13,14)^ (Chart 5). Both conditions may be related to oropharyngeal muscle hypotension, including the tongue, which, when weakened, tends to decrease the airflow, obstructing it (which causes apnea), or increasing the pressure and vibrating soft tissues (which leads to snoring)^(28)^. Both studies comprised adults in groups whose mean ages ranged from 45 to 48 years.

**Chart 5:** Summary of the main findings of the articles in individuals with sleep disorders

**Author (year of publication), country**	**Sample**	**Description of the exercises**	**Treatment frequency and duration**	**Measuring method and outcomes assessed**	**Tongue pressure**	**Results**
Ieto et al. (2015)^(13)^	39 individuals with primary snoring or mild to moderate obstructive sleep apnea.	-Pressing the tip of the tongue against the hard palate and sliding it backward.	20 repetitions of each exercise, 3x/day, for 3 months.	-Pittsburgh Sleep Quality Index.	Not assessed	-Improved sleep quality index in EG.
Brazil	EG = 19 individuals, m = 48±14 years, 11 men (oropharyngeal exercises + nasal rinsing).	-Sucking the tongue completely against the palate.		-Epworth Sleepiness Scale		-Improved personal sensation of the frequency of snoring in EG and CG.
	CG = 20 individuals, m = 45±13 years, 11 men (nasal dilator + nasal rinsing + breathing exercises)	-Lowering the tongue dorsum while keeping its tip in touch with the lower incisor.		-Snoring frequency and intensity sensation.		-Improved partner sensation of the frequency and intensity of snoring in EG.
		-Training with alternated bilateral mastication and swallowing with the tip of the tongue on the palate.		-Polysomnography		-Polysomnography with no significant changes.
		-Other exercises for the soft palate and buccinator.				
Diaféria et al. (2017)^(14)^	100 individuals with obstructive sleep apnea, m = 48.1±11.2 years, all of them males	-Tongue exercises:	3x/day, 3x/week, for 20 minutes, for 3 months.	-Epworth Sleepiness Scale.	Not assessed	-Improved Epworth Scale in EG1, EG2, and EG3 at the end of the treatment and compared with CG.
Brazil	EG1= 27 individuals (myofunctional therapy).	**(i)** Brushing the upper and lateral parts of the tongue, with the tongue on the floor of the mouth (5x each movement); **(ii)** pressing the tip of the tongue against the palate and sliding it backward (20x);		-Polysomnography (obstructive sleep apnea index, number of nocturnal wakes, and peripheral oxygen saturation).		-Improved snoring intensity and frequency in EG1, EG2, and EG3 at the end of the treatment and compared with CG.
	EG2 **=** 27 individuals (CPAP).	**(iii)** sucking the tongue completely against the palate (20x); **(iv)** tongue rotation in the vestibule (10x clockwise and 10x counterclockwise); **(v)** lowering the tongue dorsum (20x).				-Improved polysomnography in EG2 and EG3 at the beginning of the treatment and compared with CG.
	EG3 = 22 individuals (CPAP + myofunctional therapy).	-Exercises for the soft palate and buccinator.				-Improved apnea index and number of wakes in EG1 compared with CG.
	CG = 24 individuals (placebo myofunctional therapy).	-Training with mastication, swallowing, suction, and breathing.				

**Caption:** CG = Control Group; EG = Experimental Group; x = Times; m = Mean Age; CPAP = Continuous Positive Airway Pressure

Oropharyngeal exercises predominated in the studies on primary snoring and/or OSA^(13,14)^, with many parameter and frequency variations and lasting from 1 to 3 months. Tongue pressure and strength exercises were predominantly used in older adults and healthy adults. Older adults also underwent swallowing training and their training period was longer - 8 weeks on average^(21,22,26)^, while healthy adults completed training in 4 to 6 weeks^(20,23,24)^. Individuals with cancer^(25)^, usually submitted to radiotherapy or chemotherapy, mainly performed mobility exercises for the speech articulation organs (tongue, lips, and mandible). Individuals with post-stroke dysphagia^(15-17,19)^ performed tongue protrusion, retraction, lifting, and lowering for about 4 weeks. Exercises with tongue pressure against the palate were the most used in the studies, varying between isometric and isotonic exercises^(13-15,17,18,20-24,26)^, which is probably explained by their ease of performance.

The maximum anterior and/or posterior pressure were the main outcomes analyzed in the studies^(15-24,26)^, measured with the Iowa Oral Performance Instrument (IOPI). Four studies used videofluoroscopy, the gold standard method for the functional assessment of swallowing^(15,17,19,25)^. Two studies used ultrasound to assess tongue and suprahyoid muscle thickness^(20,21)^, and another two used polysomnography^(13,14)^. Other outcomes analyzed in the studies included tongue resistance^(23,24)^ using IOPI, diadochokinesia^(16)^, the percentage of correctly articulated consonants^(18)^, salivary flow rate^(22)^, impact on oral health^(27)^, sleep quality, and snoring characteristics^(13,14)^.

In general, the experimental groups (EG) had their tongue pressure increased after the treatment. Also, control groups (CG) that performed some exercises (even if different from those of EG) improved in comparison with the other CG that did not perform exercises in the studies in which they participated. Based on the concepts of exercise physiology, results were expected from the exercises because strength training recruits more motor units, increases recruitment speed and coordination, and transforms undifferentiated fibers into strength or resistance fibers^(5)^. Studies in individuals with OSA^(13,14)^ and dysphagia^(15,16,19)^ found functional improvements. Function performance benefits from improved structural strength and resistance^(5)^, although it must be pointed out that the participants in these studies also underwent functional training. Therefore, it cannot be stated whether the exercises had any effect on the function.

**Figure 2:**
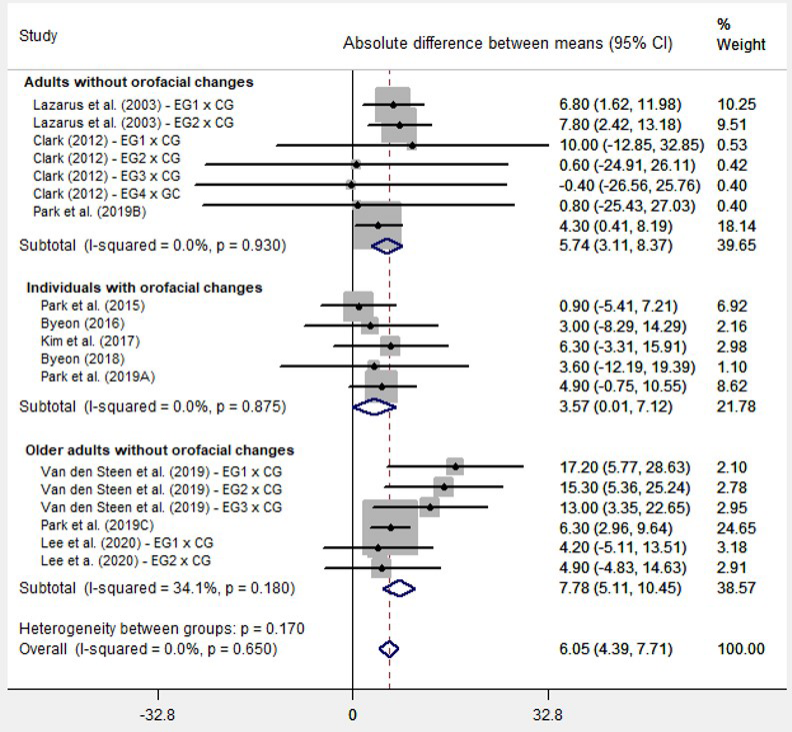
Forest Plot of the meta-analysis of the studies whose outcome was anterior tongue pressure

The first meta-analysis included 11 studies that addressed maximum anterior tongue pressure before and after the intervention in EG and CG (Figure 2). It can be noticed that the diamond at the end of the plot is located to the right and does not touch the axis, indicating that the myofunctional exercise increases the analyzed outcome (anterior tongue pressure). Cochran’s Q test found an I^2^ value of 0% and p-value = 0.650, indicating that the studies are generally homogeneous regarding the values they measured. In general, the analysis of the studies shows, in the column with the difference of means, that EG had higher values, at 6.05 kPa, with p-value < 0.001 - i.e., with a statistical significance. Some studies had more than one EG^(22,23,26)^; hence, each EG was compared with CG in an independent row. The subgroup analysis showed statistically significant differences for all subgroups, with increased pressure at 5.74 kPa among adults without orofacial changes (p < 0.001); at 7.78 kPa among older adults without orofacial changes (p < 0.001), which was the group with the best pressure gain results from the exercises; and at 3.57 kPa among individuals with orofacial changes (p = 0.049), which was the group with the least result.

**Figure 3:**
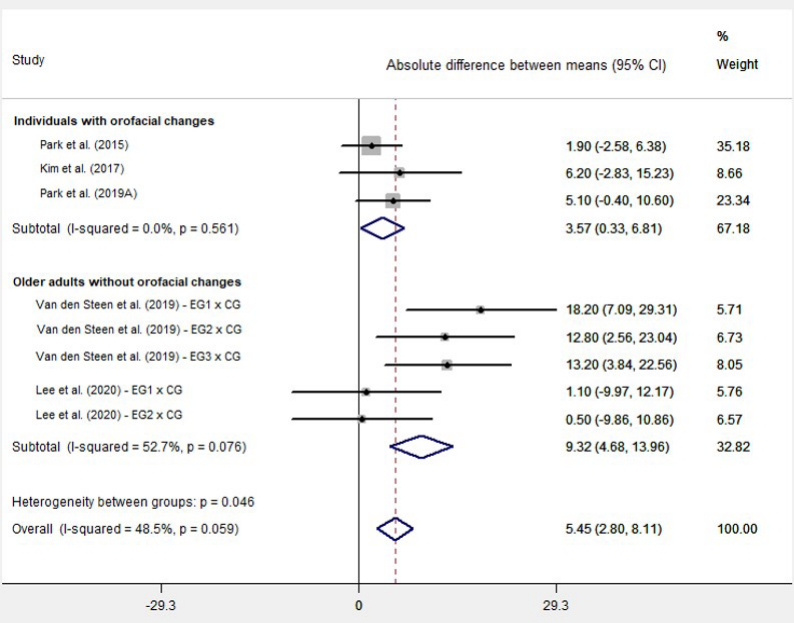
Forest Plot of the meta-analysis of the studies whose outcome was posterior tongue pressure

The second meta-analysis included five studies that addressed maximum posterior tongue pressure before and after the intervention in EG and CG (Figure 3). The diamond at the end of the plot is likewise located to the right and does not touch the axis, indicating that the myofunctional exercise increased the analyzed outcome (posterior tongue pressure). The I^2^ value of 48.5% indicates a moderate heterogeneity for these values^(29)^. The column with the difference of means shows that EG generally had higher values, at 5.45 kPa, with p < 0.001, indicating statistical evidence of differences in posterior pressure between the groups submitted to exercises and CG. Two studies had more than one EG^(20,26)^; hence, each EG was compared with CG in an independent row. The subgroup analysis showed statistically significant differences for all subgroups, with increased pressure at 9.32 kPa among older adults without orofacial changes (p < 0.001), which was the group with the best pressure gain results from the exercises; and at 3.57 kPa among individuals with orofacial changes (p = 0.049), which was the group with the least result. No study was found that assessed this outcome in adults without orofacial changes.

The group of healthy older adults probably had the best results because they initially had lower tongue pressure values (which is inherent to the aging process and is explained by the decreased muscle mass)^(30)^, combined with the absence of morphological and/or neurological changes that might hinder exercises and strength/pressure gains.

The methodological quality analysis of the studies (Table 1) had results ranging from 6 to 11 points. The highest possible score was 12 because one of the criteria the tool assesses (“Were those delivering treatment blind to treatment assignment?”) did not apply to the tongue training studies due to the nature of the intervention they addressed. Hence, the methodological quality was classified as intermediate in eight studies (57.1%) and high in six of them (42.9%). The main biases in the studies were related to participant allocation into groups. Various studies did not make it clear whether allocation had been random and blind and whether, in cases of losses to follow-up, participants were analyzed in the groups to which they had been randomly allocated. The lack of information on assessors’ blinding was another frequent bias in the studies.

**Table 1:** Analysis of the methodological quality of the studies

Author	Q1	Q2	Q3	Q4	Q5	Q6	Q7	Q8	Q9	Q10	Q11	Q12	Q13	TOTAL
Lazarus et al.^(23)^	Unclear	Unclear	Yes	No	NA	Unclear	Yes	No	Unclear	Yes	Yes	Yes	Yes	6
Clark^(24)^	Yes	Unclear	Yes	No	NA	Unclear	Yes	Yes	Yes	Yes	Yes	Yes	Yes	9
Ieto et al.^(13)^	Unclear	Unclear	Yes	No	NA	Yes	Yes	Yes	Yes	Yes	Yes	Yes	Yes	9
Park et al.^(15)^	Yes	Yes	Yes	No	NA	Yes	Yes	No	Unclear	Yes	Yes	Yes	Yes	9
Byeon^(16)^	Yes	Unclear	Yes	No	NA	Unclear	Yes	No	Unclear	Yes	Yes	Yes	Yes	7
Diaféria et al.^(14)^	Unclear	Unclear	Yes	No	NA	Yes	No	Yes	No	Yes	No	Yes	Yes	6
Kim et al.^(17)^	Yes	Unclear	Yes	No	NA	Unclear	Yes	Yes	Yes	Yes	Yes	Yes	Yes	9
Byeon^(18)^	Unclear	Unclear	Yes	No	NA	Unclear	Yes	No	Unclear	Yes	Yes	Yes	Yes	6
Van den Steen et al.^(26)^	Unclear	Unclear	Yes	No	NA	Unclear	Yes	Yes	Yes	Yes	Yes	Yes	Yes	8
Hsiang et al.^(25)^	Yes	Unclear	Yes	No	NA	Yes	Yes	Yes	Yes	Yes	No	Yes	Yes	9
Park et al.^(19)^	Yes	Yes	Yes	Yes	NA	Yes	Yes	Yes	No	Yes	Yes	Yes	Yes	11
Park et al.^(20)^	Yes	Unclear	Yes	No	NA	Yes	Yes	Unclear	Unclear	Yes	Yes	Yes	Yes	8
Park et al.^(21)^	Unclear	Unclear	Yes	No	NA	Unclear	Yes	Yes	Yes	Yes	Yes	Yes	Yes	8
Lee et al.^(22)^	Unclear	Unclear	Yes	No	NA	Unclear	Yes	Yes	No	Yes	Yes	Yes	Yes	7

**Caption:** NA = Not Applicable; Q1 = Was true randomization used for assignment of participants to treatment groups?; Q2 = Was allocation to treatment groups concealed?; Q3 = Were treatment groups similar at the baseline?; Q4 = Were participants blind to treatment assignment?; Q5 = Were those delivering treatment blind to treatment assignment?; Q6 = Were outcomes assessors blind to treatment assignment?; Q7 = Were treatment groups treated identically other than the intervention of interest?; Q8 = Was follow up complete and if not, were differences between groups in terms of their follow up adequately described and analyzed?; Q9 = Were participants analyzed in the groups to which they were randomized?; Q10 = Were outcomes measured in the same way for treatment groups?; Q11 = Were outcomes measured in a reliable way?; Q12 = Was appropriate statistical analysis used?; Q13 = Was the trial design appropriate, and any deviations from the standard RCT design (individual randomization, parallel groups) accounted for in the conduct and analysis of the trial?

**Figure 4:**
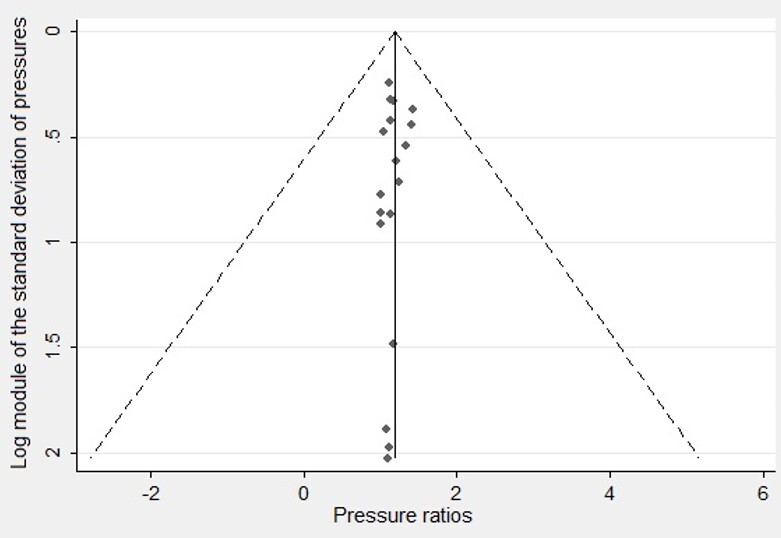
Funnel Plot of the studies whose outcome was anterior tongue pressure

**Figure 5:**
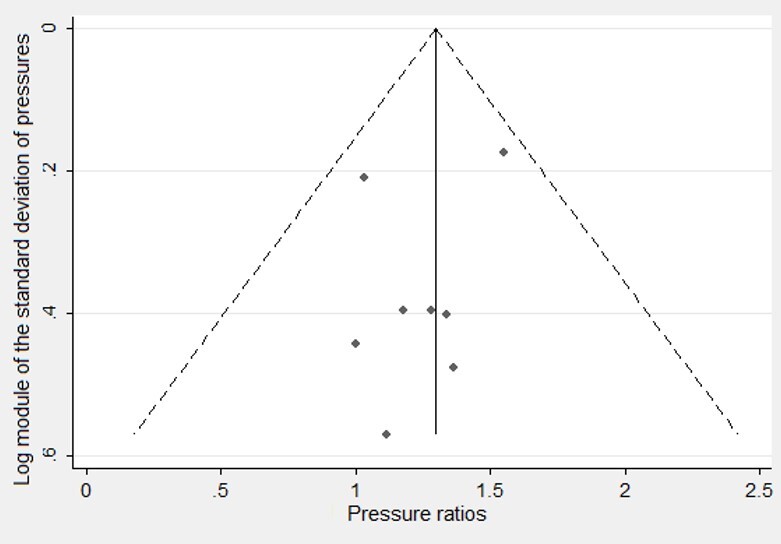
Funnel Plot of the studies whose outcome was posterior tongue pressure

The funnel plots (Figures 4 and 5) show that studies are symmetrical regarding their means and are within the 95% confidence interval lines. This demonstrates an absence of publication bias, which was corroborated by the Egger test, concerning both anterior (coefficient = 0.121; p = 0.179) and posterior pressure (coefficient = 0.621; p = 0.453).

The assessment of the quality of evidence for anterior and posterior tongue pressure began with the maximum score because the review used randomized clinical trials. Afterward, the score decreased by 2 points for the two outcomes, thus resulting in a weak certainty regarding both. In the case of anterior pressure, the score decreased because the methodological quality of more than 50% of the studies was classified as low or intermediate. As for posterior pressure, it decreased because of issues with direct evidence (absence of studies in adults that assessed this outcome) and imprecision (few participants) (Chart 6).

**Chart 6:** Quality of evidence (GRADE)

Myotherapy exercises compared with the absence of exercises in individuals older than 18 years.												
Assessment of the certainty							Number of patients		Effect		Certainty	Importance
Number of studies	Study design	Risk of bias	Inconsistency	Indirect evidence	Imprecision	Other considerations	Myotherapy exercises	Absence of exercises	Relative (95% CI)	Absolute (95% CI)		
Anterior tongue pressure (follow-up ranging from 3 to 8 weeks; assessed with IOPI)												
11	Randomized clinical trials	Very severe^a^	Not severe	Not severe	Not severe	None	252	227	-	DM 6.05 kPa higher (4.39 higher to 7.71 lower)	⨁⨁◯◯ Low	Myotherapy exercises increase anterior tongue pressure. However, the studies analyzed had methodological biases.
Posterior tongue pressure (follow-up ranging from 4 to 8 weeks; assessed with IOPI)												
5	Randomized clinical trials	Not severe	Not severe	Severe^b^	Severe^c^	None	137	126	-	DM 5.45 kPa higher (2.8 higher to 8.11 lower)	⨁⨁◯◯ Low	Myotherapy exercises increase posterior tongue pressure. However, the outcome was not assessed in adults, and there were few participants.

a Studies with moderate and low methodological quality contribute to more than 50% of the weight in the meta-analysis for this outcome;

b There was an absence of studies in adults assessing this outcome. All studies were conducted in older adults;

c Few participants had this outcome assessed

**Caption:** CI = Confidence Interval; DM = Difference of Means; IOPI = Iowa Oral Performance Instrument

This research identified that few studies have addressed this topic, especially regarding posterior tongue pressure. All included articles reported some type of benefit of tongue muscle training, with either increased anterior and/or posterior tongue pressure measures or functional improvement. The meta-analysis indicated that myofunctional exercises increased the outcomes analyzed and that older adults had the greatest benefit from this therapy. On the other hand, most of the studies had biases related to methodological quality (particularly concerning absent or inadequate randomization of participants into groups and the blinding of outcome assessors), and their quality of evidence was low. Thus, the results must be cautiously interpreted.

The limitations of this research include the search in few databases and not searching the grey literature, thus possibly failing to identify some relevant study. Another important limitation was the heterogeneity it verified regarding the sample’s characteristics and the exercise types used in the various studies. Different exercises may lead to different tongue pressure gain results^(24)^, which must be considered when interpreting the findings in this study.

## CONCLUSION

Myotherapy exercises increase anterior and posterior tongue pressure in adults. However, the quality of this evidence is low. The studies used various exercise types and training parameters. It cannot be stated whether exercises led to functional improvements.
