# Supplementary material for: The effects of lingual training: a systematic review with meta-analysis
Source: Codas. 2023 Aug 21;35(5):e20210324. doi: 10.1590/2317-1782/20232021324en (PMC10449095; doi:10.1590/2317-1782/20232021324en)
Supplement: Translation

# Translation: Efeitos do treinamento da língua: revisão sistemática com metanálise

**DOI:** 10.1590/2317-1782/20232021324pt

**Authors:** Nathalya de Faria Fonseca, Andréa Rodrigues Motta, Fernanda Campos de Freitas, Mariana Rezende Nonato, Elton Mendes Francelino, Renata Maria Moreira Moraes Furlan

**Affiliations:** 1 Universidade Federal de Minas Gerais - UFMG - Belo Horizonte (MG), Brasil.; 2 Universidade Federal da Bahia - UFBA - Salvador (BA), Brasil.

**Keywords:** Língua, Terapia por Exercício, Terapia Miofuncional, Reabilitação, Fonoaudiologia

## Abstract

**Objetivo:**

Avaliar a eficácia de exercícios mioterápicos no aumento da pressão e da força lingual. Buscou-se, de forma secundária, analisar quais os tipos de exercícios utilizados, os parâmetros de treinamento e os resultados funcionais obtidos.

**Estratégia de pesquisa:**

Revisão sistemática da literatura guiada conforme as diretrizes do protocolo Prisma.

**Critérios de seleção:**

Foram incluídos ensaios clínicos que avaliaram efeitos do treinamento muscular da língua, sem limite quanto ao idioma ou ano de publicação.

**Análise dos dados:**

Incluiu as etapas de eliminação de artigos duplicados; leitura de resumos e exclusão de estudos que não contemplaram os critérios de elegibilidade; leitura na íntegra dos artigos selecionados com extração de informações importantes, reunidas em tabela; e metanálise, realizada por meio do método do inverso da variância. A avaliação da qualidade metodológica dos estudos foi realizada pela ferramenta do *Joanna Briggs Institute*. A qualidade da evidência foi avaliada pelo Sistema *Grading of Recommendations Assessment, Development and Evaluation*.

**Resultados:**

A metanálise indicou aumento significativo da pressão máxima anterior e posterior como efeito do treinamento. O exercício mais utilizado foi pressão de língua contra o palato, mas os parâmetros de treinamento variaram entre estudos, não sendo possível afirmar que exercícios isolados promovem melhora funcional. A qualidade da evidência foi considerada baixa.

**Conclusão:**

Os exercícios mioterápicos promovem o aumento da pressão anterior e posterior da língua de indivíduos adultos, porém a qualidade dessa evidência é baixa. Há variabilidade quanto aos tipos de exercícios utilizados e parâmetros de treinamento. Não é possível afirmar que os exercícios promovem melhora funcional.

## INTRODUÇÃO

Envolvida em todas as funções do sistema estomatognático, a língua é um órgão fundamental tanto no processo de nutrição e comunicação, quanto na estabilidade da oclusão^(1)^. Sua estrutura é característica de organismos muito particulares chamados de hidróstatos musculares, como a tromba de um elefante ou os tentáculos de um polvo^(2,3)^. Trata-se de órgãos formados exclusivamente por músculos, capazes de criar e sustentar movimentos diversos, por apresentarem fibras em várias direções: longitudinal, vertical, transversal e, em alguns casos, circular^(2,3)^.

A língua é composta por músculos intrínsecos e extrínsecos e o desempenho de suas funções ocorre mediante a interação de diferentes grupos musculares, sendo que a maior parte dos movimentos requer intensa contração de vários grupos ao mesmo tempo^(4)^. Apresenta, em sua constituição morfológica, predomínio de fibras musculares dos tipos I e IIa na sua porção anterior. As fibras do tipo I são resistentes à fadiga e as do tipo IIa apresentam rápida contração. Essa combinação favorece os movimentos da fala, que são rápidos, repetitivos e não requerem muita força. Na base da língua predominam fibras do tipo IIb, que possuem maior capacidade de geração de força, o que é importante para a deglutição^(5)^.

Devido a todas essas especificidades, a aplicação da fisiologia do exercício para o tratamento das alterações relacionadas à força e/ou à resistência da língua, baseada nos mesmos princípios utilizados para os demais músculos do corpo, pode não ser uma boa opção. Esta modalidade terapêutica baseada em exercícios, chamada de mioterapia, é utilizada para a reabilitação das alterações orofaciais de origem muscular e/ou na prevenção dessas alterações; faz parte da prática fonoaudiológica em Motricidade Orofacial (MO) e tem por objetivo melhorar a força, resistência, mobilidade e coordenação^(6)^. Entretanto, além da mioterapia, e preferencialmente associada a essa, o fonoaudiólogo pode lançar mão da terapia miofuncional orofacial, que busca melhorar as estruturas e funções do sistema estomatognático por meio de treino funcional assistido^(7)^.

Há alguns anos pesquisadores têm procurado métodos para aprimorar o treinamento da musculatura da língua e muitos instrumentos e exercícios têm sido elaborados com essa finalidade^(8)^. Contudo, ao mesmo tempo em que a diversidade de exercícios promove uma ampliação das possibilidades no tratamento individualizado, afetando positivamente a sua eficácia, pode dificultar o conhecimento e o desenvolvimento dessas mesmas técnicas se não houver estudos acerca de seus efeitos. O presente artigo traz os resultados de uma investigação acerca dos efeitos do treinamento da língua a fim de refletir sobre a sua efetividade na perspectiva do cuidado fonoaudiológico.

## OBJETIVO

O objetivo desta pesquisa foi avaliar a eficácia de exercícios mioterápicos no aumento da pressão e da força lingual. Buscou-se, de forma secundária, analisar quais os tipos de exercícios utilizados, os parâmetros de treinamento (tempo de contração, número de repetições, quantidade de treinos por semana, duração do tratamento) e os resultados funcionais obtidos.

## ESTRATÉGIA DE PESQUISA

Foi realizada uma revisão sistemática da literatura, registrada na plataforma *International Prospective Register of Systematic Reviews* (PROSPERO) (CRD42021224324) e desenvolvida de acordo com as diretrizes do protocolo *Preferred Reporting Items for Systematic Reviews and Meta-Analyses* (PRISMA)^(9)^, que envolveu as seguintes etapas: elaboração da pergunta norteadora, estabelecimento de palavras-chave e de critérios de elegibilidade de artigos, seleção dos artigos e avaliação crítica desses.

A pergunta que norteou o presente estudo foi: os exercícios mioterápicos para a língua proporcionam aumento de força/pressão dessa musculatura? De forma secundária, buscou-se conhecer quais os tipos, parâmetros e efeitos funcionais dos exercícios utilizados.

Para seleção dos artigos, houve levantamento na literatura nacional e internacional, sem restrição de idioma ou do ano de publicação, utilizando-se as bases de dados Biblioteca Brasileira de Odontologia (BBO), via Biblioteca Virtual em Saúde (BVS), CINAHL, Cochrane, EMBASE, Lilacs (via BVS), Medline (via Pubmed), Scopus e Web of Science. Os descritores, obtidos nas plataformas *Medical Subject Heading* (MeSH), Descritores em Ciências da Saúde (DeCS) e *Embase Subject Headings* (Emtree), utilizados na busca foram *tongue, muscle strength, physical endurance, resistance training, exercise therapy, rehabilitation, exercise* e, os termos livres, *lingual* e *tongue strength*, assim como seus equivalentes em português e espanhol, conforme estratégias de busca apresentadas no Quadro 1. A busca, em todas as bases de dados, foi realizada no mês de setembro de 2020.

**Quadro 1:** Estratégias de busca da pesquisa por fonte

**Fonte**	**Estratégia de busca**
BVS (BBO e Lilacs)	(tongue OR lengua OR língua OR lingual) AND (“Muscle Strength” OR “Fuerza Muscular” OR “Força Muscular” OR “Physical Endurance” OR “Resistencia Física” OR “Resistência Física” OR “Força da Língua” OR “Tongue Strength” AND (“Resistance Training” OR “Entrenamiento de Resistencia” OR “Treinamento de Resistência” OR “Exercise Therapy” OR “Terapia por Ejercicio” OR “Terapia por Exercício” OR “Exercício Terapêutico” OR “Exercício de Reabilitação” OR rehabilitation OR rehabilitación OR reabilitação OR habilitação OR exercise OR “Ejercicio Físico” OR “Exercício Físico”) AND (db:(“LILACS” OR “BBO”))
CINAHL	(tongue OR lingual) AND (“muscle strength” OR “physical endurance” OR “tongue strength”) AND (“resistance training” OR “exercise therapy” OR rehabilitation OR exercise)
Cochrane	(tongue OR lingual) AND (“muscle strength” OR “physical endurance” OR “tongue strength”) AND (“resistance training” OR “exercise therapy” OR rehabilitation OR exercise)
EMBASE	tongue AND “muscle strength” OR “endurance” OR “tongue strength” AND “resistance training” OR rehabilitation OR exercise
PubMed	(tongue [MeSH Terms] OR lingual) AND (“muscle strength” [MeSH Terms] OR “physical endurance” [MeSH Terms] OR “tongue strength”) AND (“resistance training” [MeSH Terms] OR “exercise therapy” [MeSH Terms] OR rehabilitation [MeSH Terms] OR exercise [MeSH Terms])
Scopus	(ALL(tongue) OR ALL(lingual)) AND (ALL(“muscle strength”) OR ALL(“physical endurance”) OR ALL (“tongue strength”)) AND (ALL(“resistance training”) OR ALL (“exercise therapy”) OR ALL(rehabilitation) OR ALL(exercise)
Web of Science	ALL=((tongue OR lingual) AND (“muscle strength” OR “physical endurance” OR “tongue strength”) AND (“resistance training” OR “exercise therapy” OR rehabilitation OR exercise))

## CRITÉRIOS DE SELEÇÃO

Para definição dos critérios de elegibilidade, foram utilizados os elementos do PICOT: participantes (indivíduos maiores de 18 anos, sem restrição quanto ao sexo ou condição clínica); intervenção (exercícios para treino de força/pressão ou resistência de língua); comparador (indivíduos que não realizaram os exercícios propostos ou realizaram outras estratégias terapêuticas); desfechos (valores de força/pressão e desempenho em funções orofaciais); tipo de estudo (ensaios clínicos randomizados ou não). Após o estudo dos títulos e resumos, os textos compatíveis com ou que deixaram dúvidas quanto aos critérios de elegibilidade foram lidos na íntegra.

Sendo assim, com relação aos critérios de elegibilidade, foram considerados os seguintes critérios de inclusão: artigos originais de pesquisa com delineamento do tipo ensaios clínicos; com amostra composta por indivíduos acima de 18 anos que realizaram exercícios para musculatura da língua; grupo comparador composto por indivíduos que não realizaram os exercícios propostos ou realizaram outras estratégias terapêuticas; e que avaliaram como desfecho os valores de força ou pressão e/ou o desempenho em funções orofaciais. Constituíram os critérios de exclusão não abordar pelo menos um dos seguintes dados: tipo do exercício, parâmetros de realização e resultados obtidos para pelo menos um desfecho.

## ANÁLISE DOS DADOS

Após a leitura na íntegra dos artigos que contemplavam os critérios de elegibilidade, os dados foram reunidos em uma tabela que permitia o preenchimento de informações sobre: autor, ano de publicação, país onde o estudo foi realizado, características da amostra, tipo do exercício, parâmetros do treinamento, instrumentos utilizados na coleta de dados e os resultados do estudo, com ênfase para os valores de pressão ou força da língua.

A avaliação da qualidade metodológica dos estudos incluídos foi realizada por meio da ferramenta JBI *Critical Appraisal Checklist for Randomized Controlled Trials Studies* ^(10)^. O instrumento apresenta critérios para avaliação da qualidade metodológica dos estudos com três possibilidades de resposta: sim, este critério se verifica; não, este critério não se verifica e não está claro. Atribuiu-se um ponto para cada resposta sim e zero ponto para as demais respostas. Quanto maior a pontuação atingida, maior a qualidade interna e menor o risco de viés relacionado à qualidade metodológica do estudo. Determinou-se que estudos com menos de 50% das respostas positivas seriam considerados de baixa qualidade metodológica, estudos entre 50 e 75% das respostas positivas considerados de média qualidade metodológica e estudos com 75% ou mais respostas positivas seriam considerados de alta qualidade metodológica.

O viés de publicação foi analisado por meio de *funnel plot* e teste de Egger, utilizando-se o programa estatístico STATA, versão 13.0. A avaliação da qualidade da evidência foi realizada pelo Sistema Grading of Recommendations Assessment, Development and Evaluation (GRADE)^(11)^.

Todas as etapas da pesquisa foram realizadas por três pesquisadoras. A análise dos dados que determinou o cumprimento dos critérios de elegibilidade mediante a uma escala binária (sim/não) foi realizada de forma manual e independente pelas três participantes, utilizando-se uma planilha do Microsoft Excel^®^. Os artigos que obtiveram respostas sim por parte de duas avaliadoras foram incluídos no estudo. A extração dos dados foi feita, também em planilha Microsoft Excel^®^, por pelo menos uma pesquisadora e conferida por pelo menos outra pesquisadora. A análise de qualidade dos estudos foi realizada por uma pesquisadora e conferida por outra pesquisadora.

Para a metanálise, a medida de efeito da intervenção considerada foi a diferença de pressão anterior e a diferença de pressão posterior da língua entre os momentos antes e após a intervenção, a qual foi realizada por meio do método do inverso da variância no software estatístico STATA, versão 13.0. Os estudos foram analisados em conjunto, bem como de forma subdividida por condição clínica e idade.

## RESULTADOS E DISCUSSÃO

Foram localizadas, inicialmente, 526 referências nas bases consultadas sobre exercícios destinados ao treinamento da musculatura da língua (137 na Medline, sete na Lilacs, uma no BBO, 48 na CINAHL, 39 na Embase, 56 na Cochrane, 121 na Scopus e 117 na Web of Science). Após a eliminação das duplicatas ficaram 274 artigos e, com a exclusão de artigos pela leitura do resumo permaneceram 26, que foram lidos na íntegra. Após a exclusão de outros 12 artigos, que não cumpriram os critérios de elegibilidade, chegaram à fase final de inclusão para a análise 14 artigos, conforme apresentado na Figura 1.

**Figura 1:** Fluxograma com as diferentes fases da revisão baseada nas diretrizes do protocolo PRISMA. Fonte: Flow Diagram^(9)^

Ao se iniciar a análise dos estudos incluídos nesta pesquisa, nota-se que o interesse pelo tema é recente, os artigos foram publicados de 2003 a 2020. Este fato pode ser explicado pelo também recente surgimento da Motricidade Orofacial como prática fonoaudiológica regulamentada. No Brasil, por exemplo, a regulamentação da profissão de fonoaudiólogo só ocorreu em 1981, sendo que a concessão de Títulos de Especialistas, incluindo a área de Motricidade Orofacial, foi regulamentada apenas em 1996^(12)^. Os próprios instrumentos para medir força/pressão de língua, apesar de diversos, são recentes e alguns continuam em aperfeiçoamento^(8)^. Apesar disso, observa-se que a preocupação com a reabilitação das funções estomatognáticas se manifesta em diversos países. Dos 14 artigos, todos publicados em língua inglesa, dois são brasileiros^(13,14)^, sendo que os oriundos da Coreia do Sul somam o maior número, com oito textos^(15-22)^; dos Estados Unidos são dois^(23,24)^; e China^(25)^ e Bélgica^(26)^ contam com um artigo de cada nação incluídos nesta pesquisa. A faixa etária predominante das amostras compreendeu adultos jovens e idosos entre 24 e 85 anos, num quantitativo aproximado de homens e mulheres.

A maioria dos estudos foi realizada com pessoas com disfagia, quatro deles devido a acidente vascular cerebral (AVC)^(15-17,19)^ e um com pessoas com câncer em cavidade oral e/ou orofaringe submetidas à cirurgia de ressecção do tumor em pós-operatório recente^(25)^. Alterações na força/pressão da língua podem afetar tanto a fase oral quanto a fase faríngea da deglutição, sendo o emprego da força adequada crucial para uma deglutição eficiente e segura^(17)^, o que explica o número expressivo de estudos incluídos nesta pesquisa realizados com essa população. Inclui-se também nesta pesquisa um estudo realizado com pessoas com disartria pós-AVC^(18)^. As médias de idade dos indivíduos destes estudos foram semelhantes, e variaram de 56,2 a 67,3 anos. O Quadro 2 apresenta os principais achados dos estudos realizados com indivíduos com disfagia ou disartria pós-AVC e câncer de boca e/ou orofaringe.

**Quadro 2:** Resumo dos principais achados dos artigos com indivíduos com alterações orofaciais (disfagia ou disartria pós-acidente vascular cerebral e câncer de boca)

**Autor (ano de publicação), país**	**Amostra**	**Descrição do exercício**	**Frequência e duração do tratamento**	**Método de medição e desfechos avaliados**	**Pressão de língua**	**Resultados**
Park et al. (2015)^(15)^	27 adultos com disfagia pós-AVC.	-Pressão de língua contra o palato utilizando o IOPI (ponta e dorso), mantendo 80% de 1RM, por 2 s.	10 séries de 5 repetições (anterior + posterior) /dia, 5x/semana, durante 4 semanas,	-IOPI (pressão máxima anterior e posterior)	- Pressão anterior:	-Aumento na porção anterior no GE e no GC. -Aumento na porção posterior no GE.
Coreia do Sul	GE= 15 indivíduos, m=67,3±10,6 anos, 6 homens (exercícios + terapia para disfagia);		intervalo mínimo entre as repetições de 30s.	-Videofluoroscopia	GE: 18,9±6,7 kPa (antes) e 20,7±6,6 kPa (após);	-Melhora na fase oral da deglutição no GE e no GC e na fase faríngea da deglutição no GE.
	GC= 14 indivíduos, m=65,8±11,5 anos, 7 homens (terapia para disfagia)				GC: 22±5,7 kPa (antes) e 22,9±5,4 kPa (após). - Pressão posterior:	
					GE 16,2±4,7 kPa (antes) e 18,5±4,1 kPa: (após);	
					GC: 17,3±4,3 kPa (antes) e 17,7±4,36 kPa (após).	
Byeon	48 adultos com disfagia pós-AVC.	-Protrusão de língua, elevação de língua e abaixamento de língua. -Massagem na bochecha e no pescoço, fechamento e protrusão dos lábios, inflar e sugar a	Exercícios realizados por 30 minutos diários, 5x/semana durante 3 semanas.	-IOPI (pressão de elevação e pressão de protrusão, pressão de lábios e pressão de bochechas)	-Pressão anterior da língua	-Aumento da pressão de elevação anterior da língua no GE e no GC. - Melhora da diadococinesia no GE.
(2016)^(16)^	GE= 23 indivíduos, m=62,5±6,5 anos, 8 homens, (exercícios + estimulação tátil-térmica);	bochecha e protrusão, elevação e abaixamento da língua.		- Praat (diadococinesia)	GE: 20,8±13,2 kPa (antes) e 26,8±14,3 kPa (após)	
Coreia do Sul	GC= 25 indivíduos, m=64,1±7,1 anos, 6 homens (estimulação tátil-térmica).				GC: 18,5±11,5 kPa (antes) e 21,5±10,8 kPa (após).	
Kim et al. (2017)^(17)^	35 indivíduos com disfagia pós-AVC	-Exercício de contrarresistência de língua no palato (região anterior e posterior).	30x/dia, 5x/semana, durante 4 semanas.	-IOPI (pressão máxima da língua anterior e posterior)	-Pressão anterior	-Aumento na pressão anterior e posterior da língua no GE em 4 semanas.
Coreia do Sul	GE1= 18 indivíduos, m=62,2±11 anos, 11 homens (exercícios de força de língua + terapia tradicional para disfagia);			-Videofluoroscopia (avaliação funcional da deglutição - Videofluoroscopic Dysphagia Scale e escala de penetração/ aspiração de Rosenbek)	GE: 32,7±10,8 kPa (antes) e 41,9±9,5 kPa (após);	-Aumento na pressão anterior e posterior da língua no GE em relação ao GC.
	GC= 17 indivíduos, m=59,3±10,2, 8 homens (terapia tradicional para disfagia).				GC: 29,6±10,4 kPa (antes) e 32,5±10,2 kPa (após).	-Melhora na fase oral e na fase faríngea da deglutição no GE e no GC.
					-Pressão posterior	-Melhora na fase oral e na fase faríngea da deglutição no GE em relação ao GC.
					GE: 28,1±7,6 kPa (antes) e 39,1±7,8 kPa (após)	-Melhora na escala de penetração/aspiração no GE e no GC.
					GC: 26,6±9,1 kPa (antes) e 31,4±9,7 kPa (após).	
				
Byeon (2018)^(18)^	21 indivíduos com disartria pós-AVC	GE e GC: pressionar o bulbo do IOPI contra o palato, exercendo 50%, 75%, e 100% da força máxima da língua; 4 séries de 5 repetições.	30min/dia, 5x/semana, durante 4 semanas.	-IOPI (pressão máxima de língua); -Porcentagem de consoantes articuladas corretamente.	-Pressão anterior	-GE teve maior força máxima de língua do que o GC.
Coreia do Sul	GE= 10 indivíduos, m=65,85±9,23 anos (exercícios de pressão de língua + exercícios de língua) GC= 11 indivíduos, m=67,03±7,60 (exercícios de pressão de língua)	GE: (i) elevar a ponta da língua e manter 5s; (ii) elevar a ponta da língua contra uma espátula e manter 5s; (iii) lateralizar a língua e manter 5s à direita e 5s à esquerda;			GE: 10,7±9,8 kPa (antes) e 20,8±16,9 kPa (após);	-Não foram observadas diferenças na porcentagem de consoantes articuladas corretamente entre GE e GC.
		(iv) lateralizar a língua contra uma espátula e manter 5s à direita e 5s à esquerda; (v) protruir a língua por 5s e protruir a língua contra espátula por 5s.			GC: 11,4±8,1 kPa (antes) e 17,9±15,1 (após).	
Hsiang et al. (2019)^(25)^	50 adultos com câncer em cavidade oral e/ou orofaringe submetidos à cirurgia de ressecção do tumor em pós-operatório recente (48 homens, m=56,2±8,8 anos).	-Exercícios de mobilidade de língua, lábios e mandíbula: manter máxima extensão da estrutura durante 1-2s, depois relaxar.	Exercícios realizados 10x/sessão,	-Videofluoroscopia (escala de penetração/ aspiração de Rosenbek e resíduos em cavidade oral e faringe)	Não foi avaliada	-Melhora na escala de penetração/aspiração no GE. -Diminuição da quantidade de resíduos para a consistência de néctar, mel e pudim no GE.
China	GE= 25 (exercícios)	-Exercícios para resistência de língua: contrarresistência em espátula por 5s.	3 sessões/dia, durante 12 semanas.			
	GC= 25 (mudança na postura corporal e consistência alimentar)					
Park et al. (2019)^(19)^	24 adultos com disfagia pós-AVC.	-Treinamento de deglutição com esforço: pressionar a língua firmemente contra o palato, enquanto contrai a musculatura do pescoço e deglutir o mais forte possível.	Exercício realizado 10x/sessão,	-IOPI (pressão máxima anterior e pressão máxima posterior) -Videofluoroscopia (avaliação funcional da deglutição - (Videofluoroscopic Dysphagia Scale)	-Pressão anterior:	-Aumento da pressão anterior no GE e no GC. -Aumento da pressão posterior no GE e no GC. -Melhora na fase oral e na fase faríngea da deglutição no GE e no GC.
Coreia do Sul	GE= 12 indivíduos, m=66,5±9,5 anos, 6 homens (deglutição com esforço + terapia para disfagia) GC= 12 indivíduos, m=64,8±11,2 anos, 5 homens (terapia para disfagia)		3 sessões/dia,		GE: 20,8±4,3 kPa (antes) e 27,6±4,3 kPa (após);	
			5x/semana, durante 4 semanas.		GC: 21,2±5,8 kPa (antes) e 23,1±5,4 kPa (após). -Pressão posterior: GE 16,6±5,0 kPa (antes) e 23,2±5,4 kPa (após); GC: 16,7±4,4 kPa (antes) e 18,2±4,5 kPa (após).	

**Legenda:** AVC = Acidente Vascular Cerebral; GC = Grupo Controle; GE = Grupo de Estudo; IOPI = *Iowa Oral Performance Instrument*; m = Média de Idade; x = Vezes; RM = Repetição Máxima; s = Segundos

Essa atenção à força da língua e sua relação com a deglutição é mais relevante em se tratando de idosos, que apresentam tônus reduzido devido à perda de massa muscular, natural do envelhecimento, e menor força de reserva^(27)^, estando mais propensos à disfagia. Isso justifica o fato de que a metade dos estudos realizados com indivíduos sem histórico de alterações orofaciais tenha sido realizada com idosos^(21,22,26)^, enquanto a outra metade foi realizada com adultos^(20,23,24)^. Os Quadros 3 e 4 apresentam os principais achados dos estudos realizados com indivíduos sem alterações orofaciais, adultos e idosos, respectivamente.

**Quadro 3:** Resumo dos principais achados dos artigos com indivíduos adultos sem alterações orofaciais

**Autor (ano de publicação), país**	**Amostra**	**Descrição do exercício**	**Frequência e duração do tratamento**	**Método de medição e desfechos avaliados**	**Pressão de língua**	**Resultados**
Lazarus et al. (2003)^(23)^ Estados Unidos	31 indivíduos hígidos, m=26 anos.	-Pressionar a língua contra uma espátula ou contra o bulbo do IOPI nas direções esquerda, direita, protrusão e elevação, com a maior força possível e manter por 2s.	10 repetições, 5x/dia, 5x/semana, durante 4 semanas.	-IOPI (pressão máxima da língua e resistência).	-Pressão máxima anterior:	- Aumento da pressão máxima da língua no GE1 e no GE2.
	GE1**=**10 indivíduos, 2 homens (exercícios para força usando espátula).				GE1: 63,9±2,2 kPa (antes) e 72,1±2,1 kPa (após);	- Aumento da resistência da língua no GE1 e no GE2.
	GE2**=**10 indivíduos, 1 homem (exercícios para força usando IOPI).				GE2: 64,8±3,0 kPa (antes) e 74,0±2,4 kPa (após);	- Aumento da pressão máxima da língua quando comparado o resultado combinado de GE1 e GE2 em relação ao GC.
	GC**=**10 indivíduos, 5 homens (nenhuma intervenção)				GC: 69,8±5,6 kPa (antes) e 71,2±5,4 (após).	
Clark (2012)^(24)^	25 indivíduos, m=29,8 anos; 3 homens.	GE1: pressionar a língua contra o bulbo do IOPI no palato com força máxima.	1x/dia, 3x/semana, durante 4 semanas	-IOPI (pressão de elevação anterior da língua, resistência, potência e velocidade).	GE1: 65,8 kPa (antes) e 82,6 kPa (após);	-Aumento da pressão no GE1, da resistência isotônica no GE2 e da potência no GE3.
Estados Unidos	GE1= 5 indivíduos (treino de força);	GE2: pressionar o bulbo do IOPI a 50% da força máxima o maior número de vezes possível (isotônico) e pressionar o bulbo a 50% da força máxima pelo maior tempo possível (isométrico).			GE2: 65,6±15,2 kPa (antes) e 73,0±18,4 kPa (após);	-Não houve aumento da resistência isométrica no GE2 ou da velocidade no GE4.
	GE2= 5 indivíduos (treino de resistência);	GE3: repetir o fone /t/ o mais rápido possível, empurrando o bulbo a 5% da força máxima por 10s.	GE1: 5 séries de 5 repetições;		GE3: 60,2±18,0 kPa (antes) e 66,6±17,0 kPa (após);	
	GE3= 5 indivíduos (treino de potência);	GE4: repetir o fone /t/ o mais rápido possível por 10s.	GE2: 5 séries com 5% do número máximo de repetições.		GE4: 72,8±14,7 kPa (antes) e 80,4±20,1 kPa (após);	
	GE4= 5 indivíduos (treino de velocidade);		GE3: 5 séries de 10 repetições.		GC: 66,8±13,2 kPa (antes) e 73,6±10,1 kPa (após).	
	GC= 5 indivíduos (nenhuma intervenção).		GE4: 5 séries de 10s.			
Park et al. (2019)^(20)^	30 adultos hígidos GE= 15 indivíduos, m=24,5±5,3 anos, 8 homens (exercício). GC= 15 indivíduos, m=25,1±4,2 anos, 7 homens (nenhuma intervenção).	-Pressão de língua contra o palato com força máxima, realizado de forma isotônica e isométrica.	O exercício isotônico foi realizado 30x (2s) e o isométrico 3x (10s), 1x/dia, 5x/semana, durante 6 semanas.	-IOPI (pressão de língua).	-Pressão anterior	-Aumento na pressão de língua no GE. -Aumento da espessura da língua, do milo-hioideo e digástrico no GE.
Coreia do Sul				-Ultrassonografia (espessura da língua, do milohióideo e do digástrico).	GE: 52,5±4,4 kPa (antes) e 57,7±5,2 kPa (após);	-Aumento da espessura da língua no GC.
					GC: 53,8±3,0 kPa (antes) e 54,7±1,95 kPa (após).	

**Legenda:** GC = Grupo Controle; GE = Grupo de Estudo; IOPI = *Iowa Oral Performance Instrument*; m = Média de Idade; x = Vezes; s = Segundos

**Quadro 4:** Resumo dos principais achados dos artigos com indivíduos idosos sem alterações orofaciais

**Autor (ano de publicação), país**	**Amostra**	**Descrição do exercício**	**Frequência e duração do tratamento**	**Método de medição e desfechos avaliados**	**Pressão de língua**	**Resultados**
Van den Steen et al. (2019)^(26)^	60 indivíduos idosos	- Pressionar o bulbo do IOPI contra o palato em posição anterior e posterior:	60x anterior e 60x posterior, divididos em 24 séries de 5 repetições com 30s de descanso entre elas, por 3 dias não consecutivos por semana, durante 8 semanas.	-IOPI (pressão máxima anterior e posterior) no início do estudo, após 4 e 8 semanas de treinamento.	-Pressão máxima anterior:	-Todos os três grupos de estudos tiveram aumento da pressão em 4 semanas e em 8 semanas.
Bélgica	GE1= 15 indivíduos, m=79 anos, 7 homens	GE1: a 100% de 1RM;			GE1: 36,9 ± 9,1 kPa (antes) e 59,4±12,6 kPa (8 semanas)	-Pressão máxima foi maior para todos os GE em comparação com o GC.
	GE2= 16 indivíduos, m=81 anos, 7 homens	GE2: a 80% de 1RM;			GE2: 34,1 ± 8,0 kPa (antes) e 54,7±7,7 kPa (8 semanas)	
	GE3= 16 indivíduos, m=77 anos, 3 homens	GE3: a 60% de 1RM;			GE3: 35,3 ± 6,8 kPa (antes) e 53,6±7,31 kPa (8 semanas)	
	GC= 13 indivíduos	GC: pressionar o bulbo do IOPI entre os lábios.			GC: 39,2 ± 9,9 kPa (antes) e 44,5kPa± 11,7 (8 semanas)	
					-Pressão máxima posterior:	
					GE1: 30,2±8,3 kPa (antes) e 52,7±12,3 kPa (8 semanas)	
					GE2: 34,0±7,6 kPa (antes) e 51,1±9,9 kPa (8 semanas)	
					GE3: 32,8±4,4 kPa (antes) e 50,3±8,1 kPa (8 semanas)	
					GC: 34,6±8,7 kPa (antes) e 38,9±12,3 kPa (8 semanas)	
Park et al. (2019)^(21)^	40 indivíduos idosos.	-Pressão de língua contra o palato, com força a 70% 1RM, realizado de forma isotônica e isométrica.	O exercício isotônico foi realizado 30x e o isométrico 3x (30s), 3x/dia. Não foi especificada a duração do treinamento.	-IOPI (pressão de língua)	-Pressão anterior	-Aumento da pressão de língua no GE e da espessura da língua, milo-hióideo e digástrico.
Coreia do Sul	GE= 20 indivíduos, m=69,5±4,3; 10 homens (exercício);			-Ultrassonografia (espessura da língua, do milohióideo e do digástrico).	GE: 37,1±3,5 kPa (antes) e 43,9±4,9 kPa (após);	-Ausência de alteração na pressão de língua ou na espessura no GC.
	GC= 20 indivíduos, m=68,4±3,9 anos; 11 homens (nenhuma intervenção).				GC: 36,6±3,3 kPa (antes) e 37,1±3,4 kPa (após).	
Lee et al. (2020)^(22)^	74 idosos (m=75 anos)	GE1: (i) deglutir saliva sem colocar a língua pra fora; (ii) deglutir saliva com força sem colocar a língua pra fora; (iii) deglutir saliva com cerca de um terço da língua para fora; e	3x/dia, 3x/semana durante 8 semanas.	-IOPI (pressão anterior e posterior da língua e pressão labial)	-Pressão anterior	-Aumento da pressão da região anterior e posterior de língua no GE1.
Coreia do Sul	GE1= 30 indivíduos, 3 homens (deglutição com controle de língua);	(iv) deglutir saliva com cerca de dois terços da língua para fora. Três repetições de cada.		-Taxa de fluxo salivar	GE1: 34,3±10,1 kPa (antes) e 38,5±13,4 kPa (após):	-Aumento da pressão anterior da língua no grupo GE2.
	GE2= 22 indivíduos, 3 homens (treinamento de resistência com pressão de língua);	GE2: pressionar o bulbo do IOPI entre a língua e o palato duro. 30 repetições.		-Pontuação do Oral Health Impact Profile-14.	GE2: 40,6±11,5 kPa (antes) e 45,5±11,0 (após);	-Aumento da taxa de fluxo salivar no GE1 e no GE2.
	GC= 22 indivíduos, 6 homens (nenhuma intervenção).				GC: 39,1±12,9 kPa (antes) e 38,4±11,1 kPa (após).	-Ausência de impacto no Oral Health Impact Profile-14 nos grupos.
					-Pressão posterior	
					GE1: 33,8±13,8 kPa (antes) e 38,1±15,0 kPa (após);	
					GE2: 41,4±11,2 kPa (antes) e 45,1±9,7 (após);	
					GC: 35,6±15,3 kPa (antes) e 38,8±12,8 kPa (após).	

**Legenda:** GC = Grupo Controle; GE = Grupo de Estudo; IOPI = *Iowa Oral Performance Instrument*; m = Média de Idade; x = Vezes; RM = Repetição Máxima; s = Segundos

Dois estudos relacionados ao ronco primário e/ou à apneia obstrutiva do sono (AOS)^(13,14)^ (Quadro 5) foram incluídos nesta pesquisa. Ambas as condições podem estar relacionadas à hipotensão da musculatura orofaríngea, incluindo a língua que, se enfraquecida, tende a reduzir a passagem de ar, aumentando a pressão e provocando vibração das partes moles - o que resulta no ronco -, ou mesmo obstruí-la, causando apneia^(28)^. Ambos foram realizados com adultos, incluindo grupos cujas médias de idade variaram entre 45 e 48 anos.

**Quadro 5:** Resumo dos principais achados dos artigos com indivíduos com distúrbios do sono

**Autor (ano de publicação), país**	**Amostra**	**Descrição do exercício**	**Frequência e duração do tratamento**	**Método de medição e desfechos avaliados**	**Pressão de língua**	**Resultados**
Ieto et al. (2015)^(13)^	39 indivíduos com ronco primário ou apneia obstrutiva do sono leve a moderada.	-Pressionar o ápice lingual contra o palato duro e deslizá-lo para trás. -Sucção completa de língua no palato. -Abaixar o dorso da língua mantendo a ponta em contato com o dente incisivo inferior.	20 repetições de cada exercício, 3x/dia, durante 3 meses.	Questionário de qualidade do sono Pittsburgh; -Escala Epworth de sonolência; -Sensação de intensidade e frequência do ronco;	Não foi avaliada	-Melhora no índice de qualidade do sono no GE. -Melhora na sensação do indivíduo quanto à frequência do ronco no GE e no GC. -Melhora na sensação do parceiro quanto à frequência e intensidade do ronco no GE.
Brasil	GE=19 indivíduos, m=48±14 anos, 11 homens (exercícios orofaríngeos + lavagem nasal). GC= 20 indivíduos, m=45±13 anos, 11 homens (dilatador nasal + lavagem nasal + exercícios respiratórios)	-Treino da mastigação bilateral alternada e deglutição com ponta de língua no palato.		- Polissonografia		-Polissonografia sem alterações significativas.
		-Outros exercícios para palato mole e bucinador.				
Diaféria et al. (2017)^(14)^	100 indivíduos com apneia obstrutiva do sono, m=48,1±11,2 anos, todos do sexo masculino. GE1= 27 indivíduos (terapia miofuncional); GE2**=** 27 indivíduos (CPAP); GE3= 22 indivíduos (CPAP + terapia miofuncional);	-Exercícios para língua:	3x/dia, 3x/semana, durante 20 minutos, por 3 meses.	-Escala de sonolência Epworth	Não foi avaliada	-Melhora na escala Epworth no GE1, no GE2 e no GE3 ao final do tratamento e comparado ao GC.
Brasil	GC= 24 indivíduos (terapia miofuncional placebo).	**(i)** escovar as partes superior e laterais da língua, com a língua no assoalho oral (5x cada movimento); **(ii)** pressionar a ponta da língua no palato e desliza-la para trás (20x);		-Polissonografia (índice de apneia obstrutiva do sono, número de despertares noturnos e saturação periférica de oxigênio).		-Melhora na intensidade e frequência do ronco no GE1, no GE2 e no GE3 ao final do tratamento e comparado ao GC.
		**(iii)** sucção completa de língua no palato (20x); **(iv)** rotação de língua no vestíbulo (10x sentido horário e 10x anti-horário); **(v)** abaixamento de dorso de língua (20x). -Exercícios para palato mole e bucinador. -Treino de mastigação, deglutição, sucção e respiração.				-Melhora na polissonografia no GE2 e GE3 ao início do tratamento e comparado ao GC.
						-Melhora no índice de apneia e número de despertares no GE1 comparado ao GC.

**Legenda:** GC = Grupo Controle; GE = Grupo de Estudo; x = Vezes; m = Média de Idade; CPAP = *Continuous Positive Airway Pressure*

Nos estudos relacionados ao ronco primário e/ou AOS^(13,14)^ predominaram os exercícios orofaríngeos, com diversas variações de parâmetros e frequência e duração de um a três meses. Exercícios de pressão e fortalecimento de língua foram realizados predominantemente por idosos e adultos hígidos, sendo que os idosos realizaram também treino de deglutição, além de permanecerem em treinamento por mais tempo, em média oito semanas^(21,22,26)^, enquanto os hígidos completavam o treinamento entre quatro e seis semanas^(20,23,24)^. Os indivíduos com câncer^(25)^ - geralmente submetidos à radioterapia ou quimioterapia - realizaram principalmente exercícios para mobilidade dos órgãos fonoarticulatórios (língua, lábios e mandíbula). Indivíduos com disfagia pós-AVC^(15-17,19)^ realizaram protrusão, retração, elevação e abaixamento de língua por um período aproximado de quatro semanas. Observou-se que o exercício mais realizado dentre os estudos analisados foi o de pressão de língua contra o palato, variando entre isométrico e isotônico^(13-15,17,18,20-24,26)^, o que provavelmente se explica pela facilidade de execução do exercício.

Os principais desfechos analisados pelos estudos foram a pressão máxima anterior e/ou posterior^(15-24,26)^, mensuradas utilizando o *Iowa Oral Performance Instrument* (IOPI). A videofluoroscopia, método padrão ouro para a avaliação funcional da deglutição, foi utilizada em quatro estudos^(15,17,19,25)^. A ultrassonografia para avaliação da espessura da língua e musculatura suprahioidea^(20,21)^, bem como a polissonografia^(13,14)^ foram realizadas em dois estudos cada. Outros desfechos analisados foram a resistência da língua^(23,24)^ utilizando-se o IOPI, diadococinesia^(16)^, porcentagem de consoantes articuladas corretamente^(18)^, taxa de fluxo salivar^(22)^, impacto na saúde oral^(27)^, qualidade do sono e características do ronco^(13,14)^.

De modo geral, os grupos de estudo apresentaram aumento da pressão da língua após o tratamento e o grupo controle que realizou algum exercício (mesmo que fosse diferente do grupo de estudo) mostrou melhoras em relação aos outros grupos controle que não fizeram exercícios nos estudos dos quais participaram. Baseado nos conceitos de fisiologia do exercício, era de se esperar que a realização de exercícios apresentasse resultados, considerando que o treino de força promove recrutamento de mais unidades motoras, aumenta a velocidade e coordenação do recrutamento e transforma fibras indiferenciadas em fibras de força ou resistência^(5)^. Melhora nos aspectos funcionais foi verificada nos estudos com indivíduos com AOS^(13,14)^ e disfagia^(15,16,19)^. A realização das funções se beneficia da melhora de força e resistência das estruturas^(5)^, mas é preciso ressaltar que o treino funcional também foi realizado pelos participantes dos estudos mencionados. Portanto, não é possível avaliar se os exercícios em si promoveram qualquer efeito na função.

Onze estudos, por apresentarem a variável pressão máxima anterior da língua antes e após intervenção no grupo de estudo e no grupo controle, foram utilizados na primeira metanálise (Figura 2). Observa-se que o losango ao final do gráfico encontra-se à direita e não toca o eixo, refletindo que o exercício miofuncional resulta em aumento do desfecho analisado (pressão anterior de língua). O valor de I^2^ de 0% e o de p=0,650, no teste Q de Cochran, indicam que os estudos, no geral, são homogêneos quanto aos valores mensurados. Analisando os estudos, de forma geral, na coluna de diferença de médias, verifica-se que o grupo experimental apresentou valores maiores em 6,05 kPa, com valor de p<0,001, portanto apresentando significância estatística. Alguns estudos apresentaram mais de um grupo experimental^(22,23,26)^, por isso, cada grupo de estudo em comparação ao controle foi tratado em uma linha independente. Na análise por subgrupos, houve diferença estatisticamente significante para todos os subgrupos, com aumento de pressão em 5,74 kPa para adultos sem alterações orofaciais (p<0,001); em 7,78 kPa para idosos sem alterações orofaciais (p<0,001), sendo este o grupo com melhor resultado de ganho de pressão devido ao exercício; e em 3,57 kPa para indivíduos com alterações orofaciais (p=0,049), sendo este o grupo com menor resultado.

**Figura 2:** Forest Plot da metanálise dos estudos referentes ao desfecho pressão anterior da língua

Cinco estudos apresentaram a variável pressão máxima posterior da língua antes e após intervenção no grupo de estudo e no grupo controle e foram utilizados na segunda metanálise (Figura 3). Observa-se que o losango ao final do gráfico também se encontra à direita e não toca o eixo, refletindo que o exercício miofuncional resultou em aumento do desfecho analisado (pressão posterior de língua). O valor de I^2^ de 48,5% indicou heterogeneidade moderada para estes valores^(29)^. Na coluna de diferença de médias, observa-se que o grupo experimental, no geral, apresentou valores maiores em 5,45 kPa, com p<0,001, mostrando evidência estatística de diferença de pressão posterior entre os grupos submetidos aos exercícios e o grupo controle. Dois estudos apresentaram mais de um grupo experimental^(20,26)^, por isso cada grupo de estudo em comparação ao controle foi tratado em uma linha independente. Na análise por subgrupos, houve diferença estatisticamente significante para todos, com aumento de pressão em 9,32 kPa para idosos sem alterações orofaciais (p<0,001), sendo este o grupo com melhor resultado de ganho de pressão devido ao exercício; e em 3,57 kPa para indivíduos com alterações orofaciais (p=0,049), sendo este o grupo com menor resultado. Não foram encontrados estudos que avaliaram este desfecho em adultos sem alterações orofaciais.

**Figura 3:** Forest Plot da metanálise dos estudos referentes ao desfecho pressão posterior da língua

Os melhores resultados observados no grupo de idosos hígidos provavelmente ocorreram por apresentarem, inicialmente, menores valores de pressão lingual (o que é inerente ao processo de envelhecimento e explicado pela redução de massa muscular^(30)^) aliado à ausência de alterações morfológicas e/ou neurológicas que poderiam dificultar a realização do exercício e o ganho de força/pressão.

A análise da qualidade metodológica dos estudos (Tabela 1) indicou resultados que variaram entre 6 e 11 pontos, de um total de 12, considerando que um dos critérios avaliados pela ferramenta não se aplicava aos estudos do treinamento de língua: o cegamento dos pesquisadores que acompanhavam os exercícios, o qual não era possível pela natureza da intervenção estudada. Sendo assim, a qualidade metodológica foi classificada como média em oito (57,1%) estudos e alta em seis (42,9%). Os principais vieses dos estudos estiveram relacionados à alocação dos participantes nos grupos, sendo que em vários artigos não esteve claro se a alocação havia sido realizada de forma aleatória e cega e se, nos casos de perdas de seguimento, os participantes haviam sido analisados nos grupos aos quais foram randomizados. A falta de informação quanto ao cegamento dos avaliadores também constituiu viés frequente nos estudos.

**Tabela 1:** Análise da qualidade metodológica dos estudos

Autor	Q1	Q2	Q3	Q4	Q5	Q6	Q7	Q8	Q9	Q10	Q11	Q12	Q13	TOTAL
Lazarus et al^(23)^	Não está claro	Não está claro	Sim	Não	NA	Não está claro	Sim	Não	Não está claro	Sim	Sim	Sim	Sim	6
Clark^(24)^	Sim	Não está claro	Sim	Não	NA	Não está claro	Sim	Sim	Sim	Sim	Sim	Sim	Sim	9
Ieto et al.^(13)^	Não está claro	Não está claro	Sim	Não	NA	Sim	Sim	Sim	Sim	Sim	Sim	Sim	Sim	9
Park et al.^(15)^	Sim	Sim	Sim	Não	NA	Sim	Sim	Não	Não está claro	Sim	Sim	Sim	Sim	9
Byeon^(16)^	Sim	Não está claro	Sim	Não	NA	Não está claro	Sim	Não	Não está claro	Sim	Sim	Sim	Sim	7
Diaféria et al.^(14)^	Não está claro	Não está claro	Sim	Não	NA	Sim	Não	Sim	Não	Sim	Não	Sim	Sim	6
Kim et al.^(17)^	Sim	Não está claro	Sim	Não	NA	Não está claro	Sim	Sim	Sim	Sim	Sim	Sim	Sim	9
Byeon^(18)^	Não está claro	Não está claro	Sim	Não	NA	Não está claro	Sim	Não	Não está claro	Sim	Sim	Sim	Sim	6
Van den Steen et al.^(26)^	Não está claro	Não está claro	Sim	Não	NA	Não está claro	Sim	Sim	Sim	Sim	Sim	Sim	Sim	8
Hsiang et al.^(25)^	Sim	Não está claro	Sim	Não	NA	Sim	Sim	Sim	Sim	Sim	Não	Sim	Sim	9
Park et al.^(19)^	Sim	Sim	Sim	Sim	NA	Sim	Sim	Sim	Não	Sim	Sim	Sim	Sim	11
Park et al.^(20)^	Sim	Não está claro	Sim	Não	NA	Sim	Sim	Não está claro	Não está claro	Sim	Sim	Sim	Sim	8
Park et al.^(21)^	Não está claro	Não está claro	Sim	Não	NA	Não está claro	Sim	Sim	Sim	Sim	Sim	Sim	Sim	8
Lee et al.^(22)^	Não está claro	Não está claro	Sim	Não	NA	Não está claro	Sim	Sim	Não	Sim	Sim	Sim	Sim	7

**Legenda:** NA = Não se Aplica; Q1 = A alocação dos participantes aos grupos de tratamento foi verdadeiramente aleatória?; Q2 = A alocação aos grupos foi cega?; Q3 = Os grupos de tratamento eram comparáveis no início do estudo?; Q4 = Foi ocultada aos participantes a atribuição do tratamento?; Q5 = Foi ocultado aos responsáveis por aplicar o tratamento qual o grupo a que estavam alocados os participantes?; Q6 = Foi ocultado aos avaliadores dos resultados o grupo a que estavam alocados os participantes?; Q7 = Os diferentes grupos do estudo foram tratados de forma idêntica, com exceção da intervenção referida?; Q8 = O follow-up foi completado, e se não, foi abordado o uso de estratégias para colmatar a sua ausência?; Q9 = Os participantes foram analisados nos grupos aos quais foram randomizados?; Q10 = Os resultados foram avaliados da mesma forma para todos os grupos?; Q11 = Os resultados foram medidos de forma confiável?; Q12 = Foi utilizada análise estatística apropriada?; Q13 = O desenho de estudo é apropriado ao tópico em análise, e foi evidenciado algum desvio do desenho padrão de um ensaio clínico randomizado durante as fases de desenvolvimento ou análise?

De acordo com os gráficos funnel plot (Figuras 4 e 5), é possível observar que os estudos estão simétricos em relação à média e dentro das retas do intervalo de confiança de 95%. Isso indica evidência de ausência de viés de publicação, o que foi corroborado pelo teste de Egger, tanto para a pressão anterior (coeficiente=0,121; p=0,179) quanto para a posterior (coeficiente=0,621; p=0,453).

**Figura 4:** Funnel Plot dos estudos referentes ao desfecho pressão anterior da língua

**Figura 5:** Funnel Plot dos estudos referentes ao desfecho pressão posterior da língua

A avaliação da qualidade da evidência, para os desfechos pressão anterior e pressão posterior da língua, foi iniciada com pontuação máxima, visto que a revisão utiliza ensaios clínicos randomizados. Posteriormente, houve o rebaixamento em dois pontos para ambos os desfechos. Sendo assim, a certeza foi considerada fraca para ambos os desfechos. No caso da pressão anterior, o rebaixamento se deu pelo fato de que mais de 50% dos estudos tiveram qualidade metodológica classificada como baixa ou média. Já no caso da pressão posterior, o rebaixamento foi realizado por problemas na evidência direta (ausência de estudos com adultos que avaliaram este desfecho) e imprecisão (número de participantes reduzido) (Quadro 6).

**Quadro 6:** Qualidade da evidência (GRADE)

Exercício mioterápico comparado a ausência de exercício para indivíduos maiores de 18 anos												
Avaliação da certeza							Número de pacientes		Efeito		Certeza	Importância
Número de estudos	Delineamento do estudo	Risco de viés	Inconsistência	Evidência indireta	Imprecisão	Outras considerações	Exercício mioterápico	Ausência de exercício	Relativo (95% IC)	Absoluto (95% IC)		
Pressão anterior da língua (seguimento: variação 3 semanas para 8 semanas; avaliado com: IOPI)												
11	ensaios clínicos randomizados	muito grave^a^	não grave	não grave	não grave	nenhuma	252	227	-	DM 6,05 kPa mais alto (4,39 mais alto para 7,71 mais alto)	⨁⨁◯◯ Baixa	Exercícios mioterápicos promovem o aumento da pressão anterior da língua. Porém, os estudos analisados apresentam vieses metodológicos.
Pressão posterior da língua (seguimento: variação 4 semanas para 8 semanas; avaliado com: IOPI)												
5	ensaios clínicos randomizados	não grave	não grave	grave^b^	grave^c^	nenhuma	137	126	-	DM 5,45 kPa mais alto (2,8 mais alto para 8,11 mais alto)	⨁⨁◯◯ Baixa	Exercícios mioterápicos promovem aumento da pressão posterior da língua. Porém, o desfecho não foi avaliado em adultos e o número de participantes foi reduzido.

a Os estudos com qualidade metodológica moderada e baixa contribuem com mais de 50% do peso da metanálise para este desfecho;

b Ausência de estudos com adultos que avaliaram este desfecho. Todos os estudos foram realizados com idosos;

c Número de participantes para os quais foram avaliados este desfecho reduzido

**Legenda:** IC = Intervalo de Confiança; DM = Diferença de Médias; IOPI = *Iowa Oral Performance Instrument*

Esta pesquisa identificou que poucos são os estudos realizados sobre o tema, especialmente no que diz respeito à pressão posterior da língua. Todos os artigos incluídos mostraram algum tipo de benefício do treinamento da musculatura da língua, quer seja o aumento da medida de pressão anterior e/ou posterior da língua quer seja a melhora funcional. A metanálise indicou que o exercício miofuncional resultou no aumento dos desfechos analisados, sendo que os indivíduos idosos foram os que mais se beneficiaram da terapêutica. Contudo, a maioria dos estudos incluídos apresentou vieses relacionados à qualidade metodológica, principalmente quanto à falta ou inadequação da randomização dos participantes nos grupos e cegamento dos avaliadores dos desfechos, e a qualidade da evidência foi considerada baixa. Portanto, é necessária cautela na interpretação dos resultados.

Como limitações da presente pesquisa, tem-se a quantidade reduzida de bases utilizadas na busca e a não investigação da literatura cinzenta, o que pode ter ocasionado a não identificação de algum estudo relevante. Outra importante limitação é a heterogeneidade verificada, tanto relacionada às características da amostra quanto aos tipos de exercícios empregados nas diferentes pesquisas. Exercícios diferentes podem gerar resultados distintos no ganho de pressão da língua^(24)^ e isso deve ser considerado na interpretação dos achados deste estudo.

## CONCLUSÃO

Os exercícios mioterápicos promovem o aumento da pressão anterior e posterior da língua de indivíduos adultos; porém, a qualidade dessa evidência é baixa. Há variabilidade quanto aos tipos de exercícios utilizados e parâmetros de treinamento. Não é possível afirmar que os exercícios promovem melhora funcional.
